# Prevalence, Clinical Features, and In-hospital Outcome of Fatty Liver Disease in Acute Aortic Dissection: A Single-Center Retrospective Study

**DOI:** 10.3389/fcvm.2021.698285

**Published:** 2021-08-13

**Authors:** Yifan Zuo, Xin Cai, Zhiwei Wang, Zhipeng Hu, Zhiyong Wu, Min Zhang, Anfeng Yu, Liang Liu, Yun Xing

**Affiliations:** ^1^Department of Cardiovascular Surgery, Renmin Hospital of Wuhan University, Wuhan, China; ^2^Department of Cardiology, Renmin Hospital of Wuhan University, Wuhan, China

**Keywords:** aortic dissection, fatty liver disease, outcome, age, environmental parameter, gender

## Abstract

**Background and Aims:** Fatty liver disease (FLD) has emerged as a major public issue in China. We aim to investigate prevalence, clinical features, and in-hospital outcome of FLD in acute aortic dissection (AAD) patients.

**Methods:** Data of 379 AAD patients from 2017 to 2019 at Renmin hospital of Wuhan University was retrospectively collected and divided according to age and FLD absence. Propensity score matching was used for minimal confounding. We compared their physical environmental parameter of onset, clinical features, and in-hospital outcome.

**Results:** The mean age was 52.0 ± 11.5 years in type A and 55.1 ± 11.4 in type B. 25.0% of type A and 19.2% of type B AAD patients had FLD. Logistic regression indicated a negative association between FLD and age, both in type A [unadjusted odds ratio (OR) 0.958 (per 1 year), 95% confidence interval (CI) 0.930–0.988, *p* = 0.0064] and type B [unadjusted OR 0.943 (per 1 year), 95% CI 0.910–0.978, *p* = 0.0013]. After matching, type A with FLD had onset with a lower air quality index (AQI) of 68.5 [interquartile range (IQR) 46.0–90.0] and a lower Pm 2.5 concentration of 36.0 μg/m^3^ (IQR 23.0–56.0) compared with non-FLD group. In Kaplan-Meier estimation, FLD was associated with higher risk of in-hospital mortality in type B AAD (*p* = 0.0297).

**Conclusion:** The prevalence of FLD in AAD decrease with age, both in type A and type B AAD. Type A AAD patients with FLD had onset with better air quality parameters compared with non-FLD group. FLD was associated with higher risk of in-hospital mortality in type B AAD.

## Introduction

Acute aortic dissection (AAD) is a disastrous, life-threatening condition, that can be associated with a very poor outcome. Aortic dissection is conventionally classified as Stanford type A or B, with the type A involving the ascending aorta (1). Hospital studies suggested an annual incidence of AAD between 3 and 5 per 100 000 (2, 3). AAD often affected male and the risk increase with age, with untreated mortality of 1–2% per hour during the first day (4), resulting in adverse events.

Currently, changes in lifestyle and rapid urbanization led to a variety of substantial health issues, such as air pollution, poor diet, and less physical activity (5, 6). As a consequence of changes in lifestyle and rapid urbanization, fatty liver disease (FLD) has emerged as a major public issue in China, that the prevalence of FLD was 29.2% according to a meta-analysis that used data from a population of 2,054,554 (7). FLD is a multisystem disease involving a variety of extra-hepatic organs and systems. Current literature indicated that a number of pathophysiological mechanisms were recognized as being associated with the increase of cardiovascular risk in FLD, including hypertension, systemic inflammation, lipid metabolism, oxidative stress, endothelial dysfunction, arterial stiffness, etc (8–12). Despite the potential risk of FLD in cardiovascular diseases (CVD) has been evaluated in numerous studies, there was a paucity of literature that evaluate FLD in aortic diseases, such as AAD. Given the rapid increase in the prevalence of FLD and the potential CVD risk of FLD, further studies to evaluate the prevalence and in-hospital outcome of FLD in AAD remains meaningful.

This study aims to investigate the prevalence of FLD in AAD, and compare the clinical features among FLD and non-FLD groups, including in-hospital outcome, physical environmental parameters, and baseline characteristic. We think that these data could benefit clinicians in understanding and preventing this disastrous condition.

## Methods

### Study Population

From 1 January 2017 to 31 December 2019, all adult patients (≥18 years) diagnosed with aortic dissection (AD) who were admitted to Renmin Hospital of Wuhan University were enlisted. Diseases codes were extracted for preliminary identification, according to the International Classification of Diseases-10 codes (I71.0–I71.9). Radiological data (e.g., Computed tomography angiography and magnetic resonance imaging) was checked for further confirmation. The predefined exclusion criteria were as follows: (1) subacute/chronic aortic dissection, defined as an onset time over 14 days at admission; (2) simple intramural hematoma; (3) traumatic AD, AD in pregnancy, iatrogenic AD, or patients with a history of previous cardiovascular surgery/aortic dissection; (4) aortic aneurysm, penetrating atherosclerotic ulcer, non-A non-B aortic dissection, or AD with congenital abnormalities (e.g., Turner syndrome); (5) AD patients refused any further examination or treatment, or patients without complete medical records available.

### Data Collection

The following baseline features were collected individually from original medical records: gender, age, symptoms, Stanford type of AAD, medical history of diabetes mellitus and hypertension, complications at admission (such as shock, acute coronary syndrome, etc.), personal history of smoking and drinking, history of cardiovascular surgery, onset date, laboratory biomarkers, electrocardiogram, imaging data such as abdominal ultrasound, treatment, in-hospital outcome.

Hyperlipoidemia was defined as follows: (1) triglyceride >1.69 mmol/L; (2) total cholesterol >5.18 mmol/L; (3) high-density lipoprotein <0.78 mmol/L; (4) low-density lipoprotein ≥3.37 mmol/L; (5) patients receiving lipid-lowering therapy, regardless of the patient's lipid level. Current smoker was defined as smoking within 2 weeks before onset, with a history of smoking over a year. Current alcohol consumption was defined as over 20 g/d alcohol intake on average within a year. Diabetes mellitus was defined as follows: (1) fasting glucose >7.0 mmol/L; (2) patients using hypoglycemic agents or insulin; (3) patients with diagnosed diabetes. Fatty liver disease was identified based on abdominal imaging, including abdominal ultrasound, abdominal computer tomography (CT), and/or abdominal magnetic resonance imaging (MRI). Acute coronary syndrome was identified based on electrocardiogram and myocardial markers, including troponin T, troponin I, CK-MB, and myoglobin. Acute renal failure was defined as creatinine >265 μmol/L without a chronic renal failure history.

The onset date and exact time were inferred according to the occurrence of acute symptoms. The historical meteorological data of onset were obtained from the China National Meteorological Information Center (http://data.cma.cn/). Air quality was measured by the daily air quality index (AQI) and concentrations of six pollutants, including fine particulate matter 2.5 (PM2.5), fine particulate matter 10 (PM10), SO2, CO, O3. AQI were calculated using daily concentrations of individual pollutant according to Ambient Air Quality Standards (GB3095-2012). Air quality data was collected from local environmental monitoring stations.

### Study Design

This retrospective study was applied with the Strengthening the Reporting of Observational Studies in Epidemiology checklist. Only patients identified as acute aortic dissection were included. The including patients were categorized into 2 groups based on their Stanford type of AAD and analyzed individually due to the different outcome. The subgroup analysis was performed based on age to investigate the prevalence of fatty liver disease and compare other clinical features in different Stanford type individually. After age subgroup analysis, clinical features, physical environmental parameters, and in-hospital outcome were compared between FLD and non-FLD group in different Stanford type individually. The in-hospital event was defined as death of all cause in hospital, including death prior to/after operation.

### Statistical Analysis

Normally distributed continuous variables were described as mean and standard deviation (SD), and non-normally distributed continuous variables median and interquartile range (IQR). Categorical variables are described as percentages. Categorical variable and in-hospital mortality were compared using χ2 tests. Logistic regression analysis was performed to evaluate the correlation. Continuous variables were compared with one-way ANOVA or Kruskal-Wallis tests, according to whether or not distributed normally. Propensity score matching was applied for confounding control. Kaplan-Meier curves was applied for compare in-hospital survival among FLD and non-FLD group. A *p* < 0.05 was considered significant. SPSS V.24.0 was utilized for statistical analyses.

### Patient and Public Involvement Statement

This study was approved by the Laboratory Animal Welfare & Ethics Commission of Renmin Hospital of Wuhan University (WDRM 20201107). Patients were not involved in the conduct of this study due to its retrospective nature. Only county-level administrative divisions were extracted for local environmental parameter collection. All identifiable information was removed when collected from the original medical records, including names and patients' ID number, to ensure the data privacy and data safety of the included patients.

## Results

### Baseline Characteristics of the Study Population

A total of 650 patients were identified as aortic dissection by aortic computed tomography angiography. One hundred ninety-two type A AAD patients and 187 type B AAD patients with complete medical records were enrolled in the study (Supplementary Figure 1). The overall prevalence of FLD was 25.0% in type A AAD and 19.2% in type B AAD. 92.2% type A AAD patients and 98.4% type B AAD patients had sudden pain as the first symptom. Hypertension was common in both type A and type B AAD, with the prevalence of 85.9 and 87.7%, respectively. Patients with type A AAD were likely younger than type B AAD (52.0 ± 11.5 vs. 55.1 ± 11.4 years, *p* = 0.0081), with higher risk of exhibiting acute coronary syndrome (13.0 vs. 1.6%, *p* < 0.0001), shock (10.9 vs. 0%, *p* < 0.0001), and anemia (24.5 vs. 14.4%, *p* = 0.0137). Current smokers accounted for 41.1% of type A AAD patients and 45.4% of type B AAD patients. 25.0% of type A AAD and 28.3% of type B AAD experienced alcohol drinking. Baseline characteristics are summarized in Table 1.

**Table 1:** Baseline characteristic of all the 379 cases.

		**Type A AD**	**Type B AD**	
**Characteristic**		**(*N* = 192, %)**	**(*N* = 187, %)**	***p*-value**
Age, mean (SD), y		52.0 (11.5)	55.1 (11.4)	0.0081^*^
Gender	Male	155 (80.7)	158 (84.5)	0.3342
	Female	37 (19.3)	29 (15.5)	
Blood type	A	59 (30.7)	55 (29.4)	0.9648
	B	41 (21.4)	44 (23.5)	
	O	77 (40.1)	74 (39.6)	
	AB	15 (7.8)	14 (7.5)	
Onset symptom	Pain	177 (92.2)	184 (98.4)	0.0149^*^
	Syncope	8 (4.2)	1 (0.5)	
	Limb/organ ischemia	7 (3.6)	2 (1.1)	
Onset season	Spring	40 (20.8)	42 (22.4)	0.9391
	Summer	33 (17.2)	29 (15.5)	
	Autumn	53 (27.6)	49 (26.2)	
	Winter	66 (34.4)	67 (35.8)	
Living area	Rural	39 (20.3)	39 (20.9)	0.8997
	Urban	153 (79.7)	148 (79.1)	
Medical history	Hypertension	165 (85.9)	164 (87.7)	0.6121
	Diabetes mellitus	8 (4.2)	8 (4.3)	0.9570
	Hyperlipidemia	90 (46.9)	98 (52.4)	0.2816
	Fatty liver disease	48 (25.0)	36 (19.2)	0.1779
	Coronary heart disease	22 (11.4)	15 (8.0)	0.2597
	Sleep apnea syndrome	5 (2.6)	4 (2.1)	1.0000
Current smoker		79 (41.1)	85 (45.4)	0.3973
Alcohol consumption		48 (25.0)	53 (28.3)	0.4619
Complications	Acute coronary syndrome	25 (13.0)	3 (1.6)	<0.0001^*^
	Shock^c^	21 (10.9)	0 (0)	<0.0001^*^
	Renal dysfunction^d^	31 (16.1)	20 (10.7)	0.1336
	Anemia^e^	47 (24.5)	27 (14.4)	0.0137^*^
	Negative D-dimer result^f^	3 (1.6)	9 (4.8)	0.0708
Environmental parameters	Average temperature (°C)	12.25 (6.50–22.00)	14.90 (7.20–22.15)	0.2208
	Temperature difference (°C)	7.70 (5.00–10.85)	8.50 (5.05–11.30)	0.3373
	AAP (hPa)	1,011.40 (1,003.35–1,019.15)	1,011.60 (1,002.75–1,020.80)	0.6298
	APD (hPa)	4.40 (3.60–5.80)	4.70 (3.60–5.90)	0.6353
	Humidity (%)	79.0 (70.0–86.0)	78.0 (70.0–87.0)	0.9734
Air quality	AQI	75.0 (54.0–104.0)	78.0 (56.0–103.5)	0.3707
	Pm 2.5 (μg/m^3^)	44.0 (28.0–61.5)	44.0 (27.0–64.0)	0.6995
	Pm 10 (μg/m^3^)	69.0 (47.0–99.0)	72.0 (45.0–100.5)	0.6922
	SO_2_ (μg/m^3^)	10.0 (6.0–13.0)	9.0 (5.0–12.5)	0.5552
	CO (mg/m^3^)	1.00 (0.80–1.30)	1.00 (0.80–1.20)	0.8670
	NO_2_ (μg/m^3^)	31.5 (23.0–44.0)	31.0 (20.0–49.5)	0.7155
	O_3_ (μg/m^3^)	70.0 (49.5–106.5)	75.0 (50.0–116.0)	0.2336
Treatment	Surgery/TEVAR	176 (91.7)	172 (92.0)	–
	Medicine	16 (8.3)	15 (8.0)	–

*AD, Aortic dissection; SD, Standard deviation; AAP, Average atmospheric pressure; APD, Atmospheric pressure difference; AQI, Air quality index; Pm2.5, Fine particulate matter 2.5; Pm10, Fine particulate matter 10; TEVAR, Thoracic endovascular aneurysm repair*.

c *Shock was defined as systolic blood pressure <80 mmHg with signs of organ hypoperfusion, or positive vasoactive agents were applied to maintain circulation condition*.

d *Renal dysfunction was defined as Creatinine concentration >140 μmol/L*.

e *Anemia was defined as a hemoglobin concentration <120 g/L for adult male or <110 g/L for adult female at sea level*.

f *A D-dimer concentration <0.5 mg/L*.

* *p < 0.05*.

### Demographic Characteristics, Laboratory Biomarkers, and In-Hospital Outcome

Table 2 summarized the baseline characteristics and laboratory examination characteristics of AAD with/without FLD in both type A and type B. Patients identified as type A AAD with FLD were usually male, with increase in hemoglobin (Hb), alanine aminotransferase (Alt), triglyceride (Tg), and low-density lipoprotein (Ldl) level, but decrease in prothrombin time-international normalized ratio (PT-INR). The FLD patients with type A AAD were younger (48.0 ± 11.2 vs. 53.3 ± 11.4 years, *p* = 0.0058), with higher proportions of alcohol consumption (35.4 vs. 20.8%, *p* = 0.0418), hyperlipidemia (66.7 vs. 40.3%, *p* = 0.0015), but lower proportion of coronary heart disease (CHD) (2.1 vs. 14.6%, *p* = 0.0185), acute coronary syndrome (ACS) (2.1 vs. 16.7%, *p* = 0.0093), and renal dysfunction (RD) (6.3 vs. 19.4%, *p* = 0.0398).

**Table 2:** Characteristic of type A and type B acute AD with/without fatty liver disease.

	**Type A AD**			**Type B AD**		
	**With fatty liver**	**Without fatty liver**		**With fatty liver**	**Without fatty liver**	
**Characteristic**	**(*N* = 48, %)**	**(*N* = 144, %)**	***p*-value**	**(*N* = 36, %)**	**(*N* = 151, %)**	***p*-value**
**Gender**						
Male	45 (93.8)	110 (76.4)	0.0083^*^	34 (94.4)	124 (82.1)	0.0664
Female	3 (6.2)	34 (23.6)		2 (5.6)	27 (17.9)	
Age, mean (SD), y	48.0 (11.2)	53.3 (11.4)	0.0058^*^	49.5 (9.2)	56.4 (11.4)	0.0008^*^
**Living area**						
Rural	5 (10.4)	34 (23.6)	0.0616	4 (11.1)	35 (23.2)	0.1688
Urban	43 (89.6)	110 (76.4)		32 (88.9)	116 (76.8)	
**Medical history and complications**						
Current smoker	24 (50.0)	55 (38.2)	0.1500	17 (47.2)	68 (45.0)	0.8126
Alcohol consumption	18 (37.5)	30 (20.8)	0.0209^*^	12 (33.3)	41 (27.2)	0.4596
Diabetes mellitus	2 (4.2)	6 (4.2)	1.0000	0 (0)	8 (5.3)	0.3572
Hyperlipidemia	32 (66.7)	58 (40.3)	0.0015^*^	26 (72.2)	72 (47.7)	0.0081^*^
Coronary heart disease	1 (2.1)	21 (14.6)	0.0185^*^	5 (13.9)	10 (6.6)	0.1716
Hypertension	43 (89.6)	122 (84.7)	0.4015	32 (88.9)	132 (87.4)	1.0000
ACS	1 (2.1)	24 (16.7)	0.0093^*^	0 (0)	3 (2.0)	1.0000
Renal dysfunction	3 (6.3)	28 (19.4)	0.0398^*^	2 (5.6)	18 (11.9)	0.3746
Anemia	7 (14.6)	40 (27.8)	0.0656	1 (2.8)	26 (17.2)	0.0267^*^
Shock	7 (14.6)	14 (9.7)	0.3500	0 (0)	0 (0)	-
**Biomarker**						
Wbc (109/L)	12.79 (10.74–15.66)	11.78 (9.70–14.12)	0.1826	12.58 ± 3.21	11.38 ± 4.16	0.0628
Hb (g/L)	137.6 ± 17.1	126.0 ± 19.9	0.0004^*^	144.5 (136.0–157.0)	135.0 (122.0–142.0)	<0.0001^*^
Plt (109/L)	166.0 (134.0–192.0)	160.0 (128.0–186.0)	0.3208	157.0 (129.0–193.0)	166.0 (135.0–205.0)	0.9399
Alt (U/L)	29.5 (22.0–44.0)	21.0 (14.5–33.5)	0.0040^*^	27.0 (17.0–50.0)	19.0 (14.0–26.5)	0.0010^*^
Alb (g/L)	40.09 ± 3.83	38.88 ± 3.62	0.0519	41.95 ± 2.81	39.97 ± 3.67	0.0028^*^
Tbil (μmol/L)	15.98 (11.34–23.34)	16.02 (11.24–21.15)	0.8164	14.06 (10.83–21.66)	16.40 (11.40–20.86)	0.7093
Tch (mmol/L)	4.18 ± 0.97	3.97 ± 0.82	0.1602	4.57 (4.12–4.98)	4.02 (3.58–4.76)	0.0006^*^
Tg (mmol/L)	1.60 (1.05–2.12)	1.07 (0.80–1.67)	0.0085^*^	1.48 (1.13–2.74)	1.05 (0.70–1.52)	0.0004^*^
Hdl (mmol/L)	0.91 (0.82–1.17)	1.00 (0.86–1.18)	0.0861	1.04 (0.82–1.21)	1.08 (0.91–1.42)	0.0612
Ldl (mmol/L)	2.33 ± 0.69	2.10 ± 0.64	0.0397^*^	2.76 ± 0.64	2.23 ± 0.72	<0.0001^*^
Hs-CRP (mg/L)	8.24 (2.69–33.11)	8.14 (2.20–35.04)	0.6495	5.06 (1.32–30.35)	6.80 (2.52–34.04)	0.4794
Cr (μmol/L)	92.0 (73.0–118.0)	88.0 (74.0–128.0)	0.6753	77.0 (68.0–90.0)	77.0 (63.0–100.0)	0.5953
PT-INR	1.02 (0.94–1.10)	1.05 (0.99–1.14)	0.0439^*^	0.96 (0.90–1.03)	1.03 (0.96–1.08)	0.0002^*^
D-dimer (mg/L)	4.42 (3.02–11.05)	6.99 (3.72–13.82)	0.2405	2.96 (1.78–4.97)	3.27 (1.90–7.54)	0.4350
**Environmental parameters**						
Average temperature (°C)	15.25 (7.10–24.10)	11.45 (6.30–20.45)	0.1040	17.35 (8.10–21.90)	14.50 (7.60–22.45)	0.4276
Temperature difference (°C)	7.65 (5.30–10.95)	7.80 (4.40–10.90)	0.8325	8.50 (5.00–13.10)	8.90 (5.20–11.45)	0.7591
AAP (hPa)	1,008.65 (1,001.55–1,015.90)	1,013.20 (1,004.60–1,019.75)	0.0617	1,009.30 (1,004.70–1,018.90)	1,012.10 (1,002.85–1,020.60)	0.4326
APD (hPa)	4.20 (3.50–5.00)	4.50 (3.65–6.05)	0.1316	4.70 (3.80–5.70)	4.60 (3.40–5.90)	0.6301
Humidity (%)	79.0 (68.5–85.0)	79.0 (71.0–86.0)	0.5140	79.0 (70.0–87.0)	76.0 (68.5–88.0)	0.9235
**Air quality**						
AQI	75.5 (51.0–101.0)	75.0 (56.5–107.5)	0.1733	85.5 (53.0–122.0)	78.0 (59.5–100.5)	0.4069
Pm 2.5 (μg/m^3^)	40.0 (25.0–56.0)	46.0 (29.0–68.0)	0.1202	37.5 (27.0–52.0)	46.0 (27.0–64.0)	0.4643
Pm 10 (μg/m^3^)	62.0 (46.0–94.0)	71.5 (51.5–101.0)	0.3926	68.0 (53.0–107.0)	75.0 (44.5–100.5)	0.8411
SO_2_ (μg/m^3^)	9.5 (6.0–14.0)	10.0 (6.0–13.0)	0.9916	9.0 (5.0–12.0)	9.0 (5.5–13.0)	0.8811
CO (mg/m^3^)	1.00 (0.80–1.30)	1.00 (0.80–1.30)	0.9904	1.10 (0.80–1.20)	1.00 (0.80–1.20)	0.5577
NO_2_ (μg/m^3^)	30.5 (23.0–44.0)	34.0 (23.0–45.0)	0.8442	31.5 (18.0–58.0)	34.0 (20.0–48.5)	0.5028
O_3_ (μg/m^3^)	70.5 (53.0–109.0)	69.5 (46.0–106.0)	0.5667	76.5 (49.0–147.0)	77.0 (50.0–114.0)	0.3338
Surgery/TEVAR	47 (97.9)	129 (89.6)	–	36 (100.0)	136 (90.1)	–
**In-hospital survival**						
Total death	11 (22.9)	34 (23.6)	0.9216	3 (8.3)	8 (5.3)	0.4454
Death prior to operation/conversion to chronic stage	1 (2.1)	12 (8.3)	0.1910	0 (0)	5 (3.3)	0.5850
Death after operation^a^	10 (21.3)	22 (17.1)	0.5205	3 (8.3)	3 (2.2)	0.1070

*SD, Standard deviation; ACS, Acute coronary syndrome; Wbc, White blood cell counts; Hb, Hemoglobin; Plt, Platelets; Alt, Alanine aminotransferase; Ast, Aspartate aminotransferase; Alb, albumin; Tbil, Total bilirubin; Tch, Total cholesterol; Tg, Triglyceride; Hdl, High-density lipoprotein; Ldl, Low-density lipoprotein; Hs-CRP, Hypersensitive C-reactive protein; Cr, Creatinine; PT-INR, prothrombin time-international normalized ratio; AAP, Average atmospheric pressure; APD, Atmospheric pressure difference; AQI, Air quality index; Pm2.5, Fine particulate matter 2.5; Pm10, Fine particulate matter 10; TEVAR, Thoracic endovascular aneurysm repair*.

a *percentage was calculated as follows: (number of death after operation) / (number of patients underwent surgery / TEVAR) × 100%*.

* *p < 0.05*.

Type B AAD patients with FLD were younger compared with patients absent from FLD (49.5 ± 9.2 vs. 56.4 ± 11.4 years, *p* = 0.0008), had a higher proportion of hyperlipidemia (72.2 vs. 47.7%, *p* = 0.0081), but a lower proportion of anemia (2.8 vs. 17.2%, *p* = 0.0267). Compared with patients absent from FLD, type B AAD with FLD had increase in Hb, Alt, albumin, total cholesterol, Tg, and Ldl, but decrease in PT-INR.

### Logistic Regression Analysis for Main Risk Factors of FLD

Univariate logistic regression analysis was applied to investigate the relationship among FLD and other risk factors, including age, gender, living area, smoking, alcohol consumption, hypertension, diabetes, and hyperlipidemia. Table 3 summarized the logistic regression analysis for type A AAD and type B AAD, respectively. Logistic regression indicated that the prevalence of FLD decreased with age in both type A and type B AAD. In addition, the results indicated the positive association between FLD and following factors in type A AAD, including male gender (Odds ratio, OR = 4.636, 95% Confidence interval, 95% CI: 1.355–15.868, *p* = 0.0145), alcohol consumption (OR = 2.280, 95% CI: 1.122–4.635, *p* = 0.0228), and hyperlipidemia (OR = 2.966, 95% CI: 1.493–5.891, *p* = 0.0019). Hyperlipidemia was found positively associated with FLD in type B AAD (OR = 2.853, 95% CI: 1.287–6.325, *p* = 0.0099), however, male gender and alcohol consumption were not associated with FLD in type B AAD. The results also indicated the negative association between male gender and age in type A AAD, but not in type B AAD. Interestingly, acute coronary syndrome (ACS) and renal dysfunction were found negatively associated with FLD in type A AAD (Table 4), but not with age, male gender, alcohol consumption and hyperlipidemia (Supplementary Table 1).

**Table 3:** Univariate logistic regression analysis for main risk factors of fatty liver disease in acute aortic dissection.

		**Type A AAD**			**Type B AAD**		
		**OR**	**95% CI**	***P*-value**	**OR**	**95% CI**	***P*-value**
**Fatty liver disease**						
Age, linear		0.958	0.930–0.988	0.0064^*^	0.943	0.910–0.978	0.0013^*^
Gender	Female	–	Reference	–	–	Reference	–
	Male	4.636	1.355–15.868	0.0145^*^	3.702	0.838–16.352	0.0842
Area	Rural	–	Reference	–	–	Reference	–
	Urban	2.658	0.975–7.246	0.0560	2.414	0.799–7.295	0.1184
Current smoker		1.618	0.838–3.125	0.1518	1.092	0.527–2.263	0.8126
Alcohol consumption		2.280	1.122–4.635	0.0228^*^	1.341	0.615–2.927	0.4606
Hypertension		1.551	0.553–4.350	0.4044	1.152	0.366–3.620	0.8092
Diabetes mellitus		1.000	0.195–5.128	1.0000	–	NA^a^	–
Hyperlipidemia		2.966	1.493–5.891	0.0019^*^	2.853	1.287–6.325	0.0099^*^
**Gender (male)**						
Age, linear		0.940	0.909–0.973	0.0004^*^	0.971	0.937–1.006	0.1052
Area	Rural	–	Reference	–	–	Reference	–
	Urban	0.716	0.275–1.859	0.4921	1.561	0.632–3.854	0.3345
Current smoker		–	NA^b^	–	6.575	2.187–19.763	0.0008^*^
Alcohol consumption		15.667	2.086–117.664	0.0075^*^	–	NA^c^	–
Hypertension		0.482	0.137–1.695	0.2550	2.164	0.773–6.059	0.1418
Diabetes mellitus		0.126	0.029–0.556	0.0062^*^	0.162	0.038–0.691	0.0139^*^
Hyperlipidemia		2.110	0.990–4.499	0.0531			
**Age, linear**						
Area	Rural	–	Reference	–	–	Reference	–
	Urban	0.981	0.951–1.011	0.2099	0.999	0.968–1.030	0.9441
Current smoker		0.968	0.943–0.993	0.0141^*^	1.007	0.981–1.033	0.6043
Alcohol consumption		0.970	0.942–1.000	0.0463^*^	1.016	0.988–1.045	0.2731
Hypertension		1.023	0.986–1.060	0.2259	0.952	0.915–0.991	0.0175^*^
Diabetes mellitus		1.003	0.943–1.067	0.9209	1.032	0.969–1.099	0.3241
Hyperlipidemia		0.982	0.958–1.007	0.1535	0.975	0.950–1.001	0.0605

*AAD, Acute aortic dissection; OR, Odds ratio; CI, Confidence interval; NA, Not available*.

a *All enlisted subjects with FLD in type B AAD were absent from diabetes mellitus*.

b *All smokers were male in type A AAD*.

c *All drinkers were male in type B AAD*.

* *p < 0.05*.

**Table 4:** Univariate logistic regression analysis for complications of fatty liver disease in acute aortic dissection.

	**B**	**S.E**.	**Wald**	***P*-value**	**OR**	**95% CI**
**Type A AAD**						
ACS	−2.241	1.035	4.687	0.0304^*^	0.106	0.014–0.809
Shock	0.461	0.496	0.862	0.3532	1.585	0.599–4.914
Renal dysfunction	−1.287	0.632	4.140	0.0419^*^	0.276	0.080–0.954
Anemia	−0.812	0.449	3.268	0.0707	0.444	0.184–1.071
**Type B AAD**						
ACS	–	–	–	0.9993	–	NA^a^
Renal dysfunction	−0.833	0.770	1.172	0.2790	0.435	0.096–1.965
Anemia	−1.985	1.037	3.666	0.5555	0.137	0.018–1.048

*AAD, Acute aortic dissection; ACS, Acute coronary syndrome; NA, not available*.

a *All enlisted subjects with ACS in type B AAD were absent from FLD*.

* *p < 0.05*.

**Figure 1:**
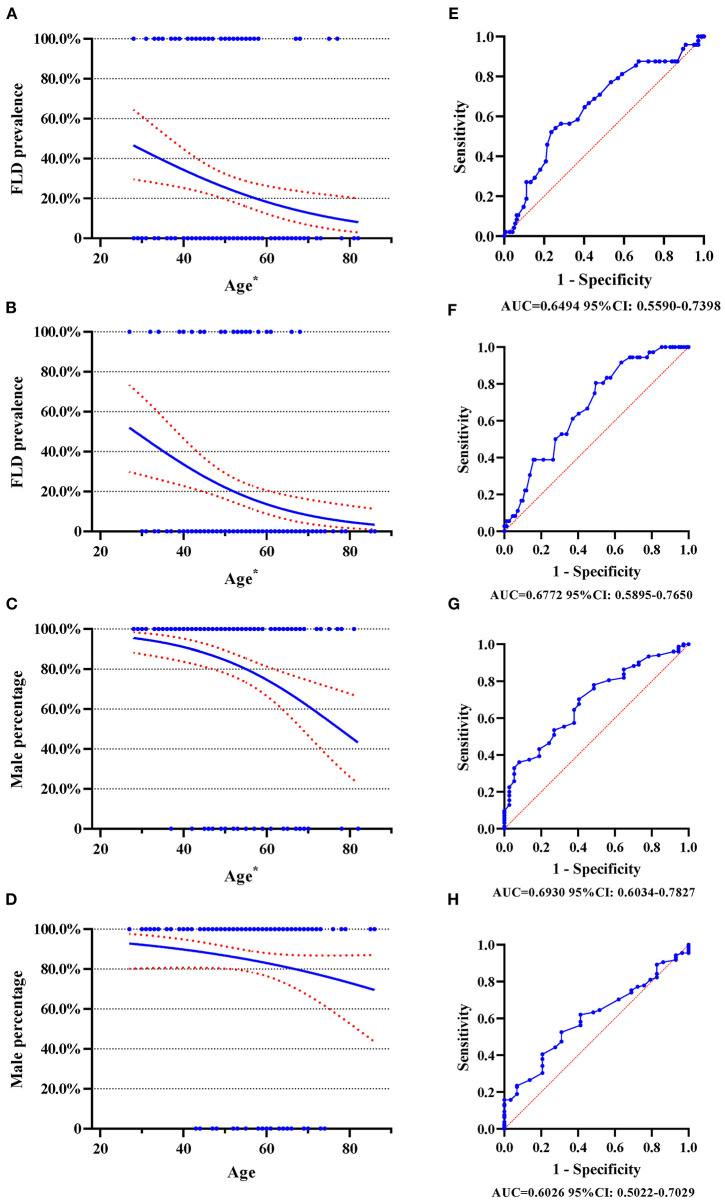
Logistic regression plot and ROC curve with 95% CI. ROC curve, receiver operating characteristic curve; FLD, fatty liver disease; AAD, acute aortic dissection. **(A)** Logistic regression plot for FLD and age in type A AAD; **(B)** Logistic regression plot for FLD and age in type B AAD; **(C)** Logistic regression plot for male gender and age in type A AAD; **(D)** Logistic regression plot for male gender and age in type B AAD; **(E)** ROC curve for FLD and age in type A AAD; **(F)** ROC curve for FLD and age in type B AAD; **(G)** ROC curve for male gender and age in type A AAD; **(H)** ROC curve for male gender and age in type B AAD. The numbers beneath each drawing represent area under curve and 95% CI. *p < 0.05.

As shown in Figure 1, the prevalence of FLD decrease with age. In type A AAD, around 40% of patients aged 30 years had FLD, while this proportion was around 20% in patients aged 60 years (Figure 1A). In type B AAD, approximately 50% of patients aged 30 years had FLD, however, this proportion decreased to about 10% for patients aged 60 years (Figure 1B). The proportion of male also decreased with age in type A AAD (Figure 1C), but this negative trend was insignificant in type B AAD (Figure 1D).

### Subgroup Analysis for Main Risk Factors of FLD

Since significant association between FLD and several risk factors as summarized in Tables 3, 4 subgroup analyses were performed subsequently based on univariate logistic regression analysis. Supplementary Table 2 summarized gender subgroups of both type A and type B AAD. Male type A AAD patients were younger, had higher proportion of alcohol consumption, smoking, but a lower proportion of diabetes. Male type B AAD patients had higher proportion of alcohol consumption, smoking, but a lower proportion of diabetes, which was similar to male type A AAD patients. 29.0% of male patients and 8.1% of female patients in type A AAD had FLD, and 21.5% of male patients and 6.9% of female patients in type B AAD had FLD. Supplementary Tables 3, 4 shown age subgroups of type A and type B AAD, respectively. The results shown the differences in FLD prevalence between different age subgroup, which was consistent with univariate logistic regression analysis mentioned above.

Supplementary Table 5 summarized alcohol consumption subgroups in type A and type B AAD. Most of drinkers were male, had a higher proportion of smoking and increase in Hb level for both type A and type B AAD. 37.5% of drinkers and 20.8% of non-drinkers in type A AAD had FLD, and 22.6% of drinkers and 17.9% of non-drinkers in type B AAD had FLD. Supplementary Table 6 shown the results of hyperlipidemia subgroups. Hyperlipidemia subgroup had increase in Tch, Tg, Hdl, and Ldl level compared with patients without hyperlipidemia in both type A and type B AAD, as expected. Hyperlipidemia subgroup had a higher prevalence of FLD (35.6% for type A and 26.5% for type B), while normolipidemic controls had a lower prevalence of FLD (15.7% for type A and 11.2% for type B).

### Clinical Features, Physical Environmental Parameters of Onset, and In-hospital Outcome After Propensity Score Matching

Since that AAD patients with FLD had different demographic characteristics compared with AAD patients absent from FLD, a propensity score matching (PSM) was applied to achieve balanced exposure groups at baseline (i.e., minimal confounding). The steps and covariates used for PSM were presented in Supplementary Material.

Baseline characteristics, clinical features, physical environmental parameters and in-hospital outcome of type A and type B AAD were summarized in Table 5. After matching, type A AAD with FLD had onset with lower AQI and Pm 2.5 levels compared with non-FLD group. Although AAD with FLD presented a higher average temperature of onset, no significant difference was found in environmental parameters between FLD group and non-FLD group. Type B AAD with FLD shared similar climatic parameters and air quality with non-FLD group. AAD with FLD had a higher rate of in-hospital death.

**Table 5:** Characteristic of acute aortic dissection with/without fatty liver disease after propensity score matching.

	**Type A AD**			**Type B AD**		
	**With fatty liver**	**Without fatty liver**		**With fatty liver**	**Without fatty liver**	
**Characteristic**	**(*N* = 40, %)**	**(*N* = 67, %)**	***p*-value**	**(*N* = 34, %)**	**(*N* = 52, %)**	***p*-value**
**Gender**						
Male	37 (92.5)	57 (85.1)	0.3631	32 (94.1)	50 (96.2)	0.6462
Female	3 (7.5)	10 (14.9)		2 (5.9)	2 (3.8)	
Age, mean (SD), y	49.4 (11.4)	50.4 (11.4)	0.6536	49.4 (9.5)	50.5 (9.9)	0.6061
**Medical history and complications**						
Current smoker	20 (50.0)	32 (47.8)	0.8226	17 (50.0)	26 (50.0)	1.0000
Alcohol consumption	12 (30.0)	16 (23.9)	0.4860	12 (35.3)	18 (34.6)	0.9485
Diabetes mellitus	2 (5.0)	2 (3.0)	0.6287	0 (0)	0 (0)	–
Hyperlipidemia	24 (60.0)	34 (50.7)	0.3526	24 (70.6)	39 (75.0)	0.6513
Coronary heart disease	1 (2.5)	1 (1.5)	1.0000	3 (8.8)	4 (7.7)	1.0000
Hypertension	36 (90.0)	58 (86.6)	0.7631	30 (88.2)	46 (88.5)	1.0000
ACS	1 (2.5)	0 (0)	0.3738	0 (0)	0 (0)	–
Renal dysfunction	3 (7.5)	14 (20.9)	0.0998	2 (5.9)	4 (7.7)	1.0000
Anemia	7 (17.5)	13 (19.4)	0.8070	1 (2.9)	2 (3.8)	1.0000
Shock	7 (17.5)	7 (10.4)	0.2953	0 (0)	0 (0)	–
**Biomarker**						
Wbc (109/L)	12.79 (10.25–15.47)	12.16 (10.30–15.64)	0.9658	12.74 ± 3.16	12.21 ± 3.88	0.5042
Hb (g/L)	135.2 ± 17.1	130.9 ± 21.9	0.2934	144.0 (136.0–156.5)	141.0 (134.0–152.0)	0.1985
Plt (109/L)	163.0 (131.0–185.0)	158.0 (130.0–184.5)	0.9264	155.0 (129.0–191.5)	179.0 (144.5–206.0)	0.3182
Alt (U/L)	29.5 (19.0–41.0)	24.0 (16.0–35.0)	0.1968	24.5 (16.5–46.5)	20.0 (16.0–28.5)	0.0550
Alb (g/L)	39.73 ± 3.76	39.23 ± 3.71	0.5108	42.01 ± 2.76	41.36 ± 3.08	0.3219
Tbil (μmol/L)	15.68 (12.46–22.64)	16.61 (11.13–20.32)	0.7346	14.76 (10.88–21.68)	18.40 (13.68–27.02)	0.2381
Tch (mmol/L)	4.17 ± 0.95	4.09 ± 0.82	0.6507	4.72 ± 0.74	4.46 ± 1.02	0.1781
Tg (mmol/L)	1.60 (1.05–1.96)	1.07 (0.77–2.04)	0.2068	1.44 (1.06–2.61)	1.23 (0.78–2.18)	0.1138
Hdl (mmol/L)	0.91 (0.82–0.98)	0.97 (0.84–1.11)	0.2840	1.04 (0.83–1.22)	0.98 (0.86–1.18)	0.9564
Ldl (mmol/L)	2.33 ± 0.67	2.14 ± 0.63	0.1481	2.78 ± 0.64	2.40 ± 0.79	0.0211
Hs-CRP (mg/L)	9.46 (2.69–33.75)	8.59 (3.58–29.76)	0.5606	5.06 (1.21–33.64)	9.48 (4.12–27.09)	0.5912
Cr (μmol/L)	91.5 (74.0–118.0)	93.0 (77.5–140.0)	0.2977	78.0 (68.0–90.5)	77.5 (64.0–99.5)	0.7404
PT-INR	1.04 (0.97–1.11)	1.03 (0.98–1.12)	0.7124	0.95 (0.90–1.04)	1.03 (0.98–1.09)	0.0029^*^
D-dimer (mg/L)	4.94 (3.08–11.05)	8.20 (3.94–15.65)	0.3282	2.96 (1.80–4.86)	2.53 (1.78–5.27)	1.0000
**Environmental parameters**						
Average temperature (°C)	15.30 (8.80–25.50)	11.60 (6.05–19.70)	0.1944	16.05 ± 9.05	13.70 ± 8.61	0.2295
Temperature difference (°C)	8.10 (5.50–10.80)	7.60 (3.55–12.40)	0.8267	7.90 (4.95–12.55)	8.55 (5.00–10.30)	0.9261
AAP (hPa)	1,007.45 (1,001.50–1,015.70)	1,013.70 (1,004.20–1,020.55)	0.1352	1,009.96 ± 13.26	1,014.77 ± 10.68	0.0675
APD (hPa)	4.25 (3.50–5.10)	4.40 (3.50–5.55)	0.4041	4.70 (3.70–5.80)	4.60 (3.65–5.75)	0.9930
Humidity (%)	77.3 ± 12.1	78.7 ± 11.4	0.5514	77.9 ± 11.9	77.8 ± 11.7	0.9713
**Air quality**						
AQI	68.5 (46.0–90.0)	77.0 (59.0–109.5)	0.0435^*^	82.0 (52.5–124.5)	80.5 (62.0–109.5)	0.8252
Pm 2.5 (μg/m^3^)	36.0 (23.0–56.0)	53.0 (29.5–75.0)	0.0276^*^	36.5 (26.5–54.0)	43.0 (22.5–67.0)	0.5568
Pm 10 (μg/m^3^)	60.0 (44.0–91.0)	78.0 (51.5–107.0)	0.2425	66.5 (51.5–106.5)	76.0 (40.0–97.0)	0.8911
SO_2_ (μg/m^3^)	9.0 (6.0–14.0)	9.0 (6.5–13.0)	0.9871	8.5 (5.0–11.0)	10.0 (6.0–12.0)	0.8384
CO (mg/m^3^)	1.00 (0.80–1.20)	1.00 (0.80–1.40)	0.3577	1.10 (0.80–1.25)	1.00 (0.80–1.30)	0.6696
NO_2_ (μg/m^3^)	30.5 (25.0–44.0)	35.0 (25.0–45.0)	0.5090	29.5 (17.0–52.5)	35.0 (16.5–51.0)	0.8389
O_3_ (μg/m^3^)	68.5 (53.0–105.0)	67.0 (50.0–109.0)	0.9563	69.5 (44.0–143.5)	74.5 (46.0–115.0)	0.4474
Surgery/TEVAR	39 (97.5)	61 (91.0)	–	34 (100.0)	48 (92.3)	–
**In-hospital survival**						
Total death	9 (22.5)	14 (20.9)	0.8450	3 (8.8)	0 (0)	0.0585
Death prior to operation/conversion to chronic stage	1 (2.5)	5 (7.5)	0.4073	0 (0)	0 (0)	–
Death after operation^a^	8 (20.0)	9 (14.8)	0.4907	3 (8.8)	0 (0)	0.0676

*SD, Standard deviation; ACS, Acute coronary syndrome; Wbc, White blood cell counts; Hb, Hemoglobin; Plt, Platelets; Alt, Alanine aminotransferase; Ast, Aspartate aminotransferase; Alb, Albumin; Tbil, Total bilirubin; Tch, Total cholesterol; Tg, Triglyceride; Hdl, High-density lipoprotein; Ldl, Low-density lipoprotein; Hs-CRP, Hypersensitive C-reactive protein; Cr, Creatinine; PT-INR, Prothrombin time-international normalized ratio; AAP, Average atmospheric pressure; APD, Atmospheric pressure difference; AQI, Air quality index; Pm2.5, Fine particulate matter 2.5; Pm10, Fine particulate matter 10; TEVAR, Thoracic endovascular aneurysm repair*.

a *percentage was calculated as follows: (number of death after operation)/(number of patients underwent surgery/TEVAR) × 100%*.

* *p < 0.05*.

**Figure 2:**
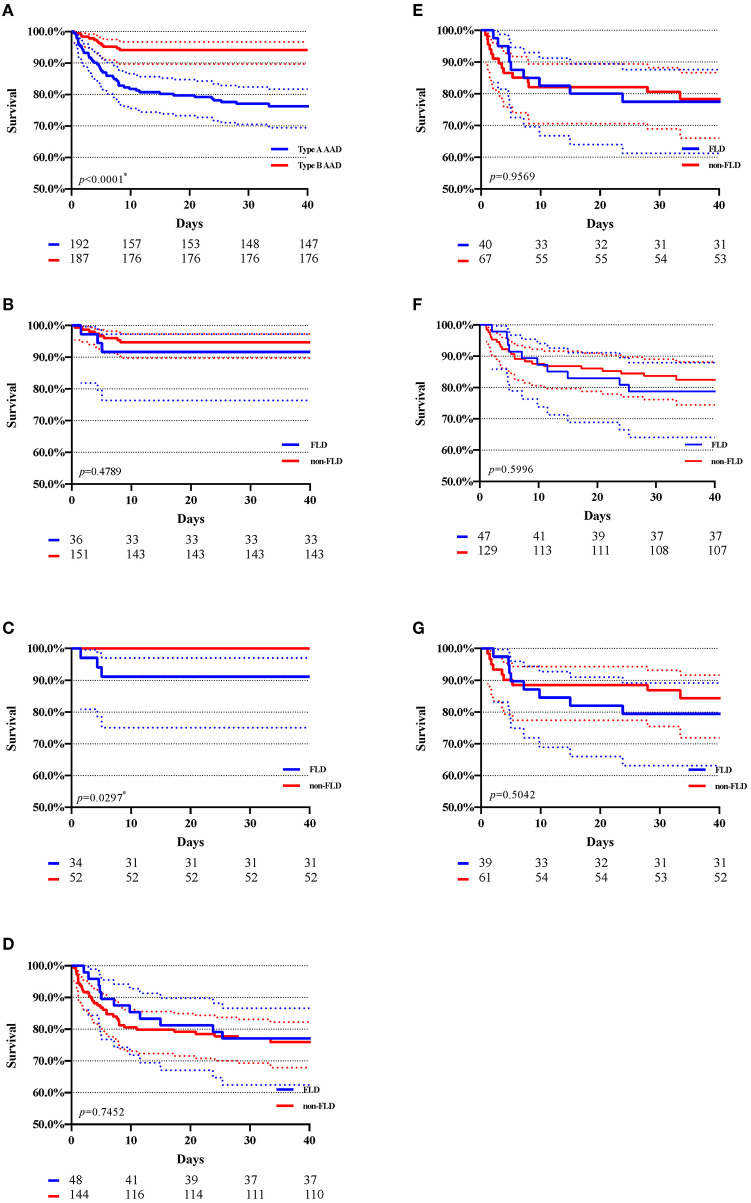
Kaplan–Meier estimation of in-hospital survival with 95% CI. AAD, acute aortic dissection. FLD, fatty liver disease. **(A)** Total in-hospital survival of type A and type B AAD; **(B)** In-hospital survival of type B AAD before matching. **(C)** In-hospital survival of type B AAD after matching; **(D)** In-hospital survival of type A AAD after matching; **(E)** in-hospital survival of type A AAD after matching; **(F)** In-hospital survival of type A AAD (patients underwent surgery) before matching; **(G)** In-hospital survival of type A AAD (patients underwent surgery) after matching; The numbers beneath each drawing represent the number of survivors in 10, 20, 30, and 40 days. *p < 0.05.

Figure 2 showed the Kaplan-Meier curves of in-hospital survival before and after matching. Figure 2A indicated that type B AAD presented a higher total in-hospital survival compared with Type A AAD (94.1% of type B subjects and 77.1% of type A subjects survived at 30 days). After matching, a significant difference revealed between the FLD and non-FLD groups in type B AAD (*p* = 0.0297). As regards in-hospital survival in type A AAD, Figures 2D,E shown Kaplan-Meire curve of total in-hospital survival with 95% CI in type A AAD before and after matching, respectively. Figures 2F,G shown Kaplan-Meier curve of surgical in-hospital survival with 95% CI in type A AAD before and after matching, respectively. Kaplan-Meier estimation indicated no significant differences among the FLD and non-FLD groups before and after matching.

## Discussion

The results of this study suggest the following. (1) The prevalence of fatty liver disease decreases with age in both type A and type B acute aortic dissection, as shown in Figure 1 and Table 3. (2) The prevalence was greater in type A acute aortic dissection patients with male gender, alcohol consumption, and hyperlipidemia. (3) The prevalence was greater in type B acute aortic dissection patients with hyperlipidemia. (4) Type A acute aortic dissection with fatty liver disease had onset with better air quality parameters, compared with cases without fatty liver disease. (5) Fatty liver disease was associated with a higher risk of in-hospital death risk in type B acute aortic dissection.

Our results confirmed that the prevalence of FLD showed a decrease with age (Figure 1) that the total prevalence of FLD was 25.0% in type A and 19.2% in type B AAD. Recently, FLD has emerged as a major public health issue in China, with a national prevalence of 29.2% and the youngest median age worldwide (7, 13). The association between FLD and various CVD has been described previously, including atherosclerosis, structural cardiac alterations, and functional cardiac abnormalities such as coronary artery disease or arterial hypertension, which might result in adverse cardiovascular events (14, 15). FLD is recognized as a risk factor of endothelial dysfunction, while insulin resistance and visceral fat, that are conferred as increased risks of CVD, are also strongly linked to fatty liver (16). Furthermore, FLD also plays a significant role in inflammation, leading to increased inflammatory factors such as high-sensitivity C-reactive protein and IL-6, which are also a predictor of CVD (16). Additionally, FLD is linked to increased arterial stiffness by several mechanisms (9–11), which is usually a consequence of aging and metabolic disorder (17). Arterial stiffness contributes to an increase in its structural vulnerability, suggesting reduced structural integrity or strength (18). Therefore, a possible hypothesis is that FLD might also be associated with aortic disease to some extent. In fact, a retrospective case-control study evaluating FLD in abdominal aortic aneurysm (AAA) reported increased prevalence of FLD, with younger mean age in FLD subgroup among the AAA patients (19), which is similar to our results. These suggest that, FLD might contribute to aortic alteration via several mechanisms, such as inflammation or structural vulnerability, resulting in an increased risk in aortic mechanical insufficiency and diseases, which explain the decreased prevalence of FLD with age in acute aortic dissection.

Previous studies have described AAD as one of the most catastrophic, life-threatening cardiovascular diseases with a typical chronobiological pattern, associated with extremely poor outcomes and a high risk of readmission (20–23). AAD was described as a high incidence during the cold period, with a peak in January (22), suggesting a potential connection between climate characteristics and the onset of AAD. The incidence of AAD was correlated to the average temperature and atmospheric pressure, as a study of 1,642 patients in two continents reported (24). Climatic changes, such as temperature, humidity, and atmospheric pressure, have the potential to provoke physiological changes on humans. During the colder periods, cold exposure can trigger several physiological responses, such as vasoconstriction, elevated blood pressure, platelet activation, or increased levels of inflammatory blood biomarkers. These acute and chronic physiological responses have potentially adverse cardiovascular effects (24–26). In fact, an increased systolic blood pressure (SBP) associated with low outdoor temperature was reported previously, with 6 mmHg higher SBP per 10°C lower outdoor temperature (27). Besides climatic changes, exposure to air pollution, such as Pm 2.5, is also associated with an increase in acute AD hospitalization and increased mortality from various cardiopulmonary diseases (28, 29). Furthermore, Pm 2.5 exposure is also linked to increased risk of metabolic disorder and chronic liver injury (30), suggesting that climatic changes and air pollution can trigger the onset of AAD by various mechanisms.

Given that FLD might contribute to aortic alteration and the typical chronobiological pattern of AAD, we compared the physical environmental parameters between FLD and non-FLD group in different Stanford type individually. After matching, the subgroup of type A AAD patients with FLD presented an onset with a relatively better environmental condition, which was usually warmer with better air quality, especially with lower AQI (IQR 46.0–90.0 vs. 59.0–109.5, *p* = 0.0435) and level of Pm 2.5 concentration (IQR 23.0–56.0 vs. 29.5–75.0, *p* = 0.0276). In contrast, no significant difference in environmental condition of onset was found before matching. Air pollutant, such as Pm 2.5, is considered important environmental risk factor for human health worldwide. Despite the mechanisms of how air pollutant, such as Pm 2.5, provokes AAD remains unclear, a time-series study in Shanghai reported an association short-term Pm 2.5 exposure and increased AAD hospitalizations recently, especially when daily Pm 2.5 concentration was over 35 μg/m^3^ (28). The low concentration of type A AAD with FLD suggests that, FLD might lead to increased susceptibility to the effects of air pollutant exposure, resulting the onset of AAD with relatively better air quality. However, no significant difference in environmental conditions was found between FLD group and non-FLD group of type B AAD. The possible reason is that type B acute AD usually presents abdominal pain or gastrointestinal discomfort, which might result in a delay of diagnosis. In fact, about 30.7% of type B aortic dissection patients (94 in 306) were identified as subacute or chronic AD, indicating that three-tenths of type B aortic dissection patients might not provide credible onset data. In type A AAD, only 4.5% (11 in 242) of type A aortic dissection patients were identified as subacute or chronic AD, where 94.8% patients (182 in 192) included in the study had an admission within 3 days after onset. These indicated the delay in diagnosis of AD might be relatively more common in type B aortic dissection. Therefore, partial climatic and air quality information might be lost in type B aortic dissection, which might conceal the difference in climatic and air quality parameters of onset between FLD group and non-FLD group in type B AAD.

In summary, we investigated the difference between subgroups with different ages of AAD patients in central China, and compared the climatic parameters of onset as well as in-hospital survival between FLD and non-FLD acute AD patients. Our results confirmed a higher prevalence of FLD in young acute AD patients, both in type A and type B AD. After PSM, lower AQI and Pm2.5 levels of onset were found in the FLD group in type A AAD compared with non-FLD group. In-hospital survival was similar among FLD and non-FLD groups in type A AAD. However, FLD was associated with a higher risk of in-hospital death risk in type B AAD. These findings may provide some new perspectives in understanding the pathogenesis of acute aortic dissection for clinicians, and conduce to better prevention of this devastating disease. Given the rapid increase in FLD prevalence and adverse cardiovascular effects (7, 14, 16), further studies are necessary to understand the underlying pathophysiological mechanisms between FLD and aortic diseases, which could be beneficial to the prevention and treatment of this catastrophic disease.

This study has some limitations. First, this study was single-center retrospective designed, that main population involved was Han people from central China. Therefore, cautions should be taken if the results of this study are extrapolated to other populations or regions. Second, the study was based on data from hospital-based patients rather than population-based samples. Given the high rate of out-of-hospital deaths in AAD, some patients might fail in receiving timely medical assistance. Third, abdominal imaging data, such as ultrasound, CT and/or MRI, were the main investigation to confirm the presence of FLD, which might underestimate the prevalence of FLD in acute AD patients. Further investigation based on other populations and regions is necessary.

## Data Availability Statement

The raw data supporting the conclusions of this article will be made available by the authors, without undue reservation.

## Ethics Statement

This study was approved by the Laboratory Animal Welfare & Ethics Commission of Renmin Hospital of Wuhan University (WDRM 20201107). Informed consent was not required for this study.

## Author Contributions

ZWa: guarantor of the article. ZWa, YZ, and XC: conception/design. YZ, XC, ZH, ZWu, MZ, AY, LL, and YX: collection and/or assembly of data, data analysis, and interpretation. YZ, XC, ZWa, ZH, ZWu, MZ, AY, LL, and YX: manuscript writing and final approval of manuscript. All authors contributed to the article and approved the submitted version.

## Conflict of Interest

The authors declare that the research was conducted in the absence of any commercial or financial relationships that could be construed as a potential conflict of interest.

## Publisher's Note

All claims expressed in this article are solely those of the authors and do not necessarily represent those of their affiliated organizations, or those of the publisher, the editors and the reviewers. Any product that may be evaluated in this article, or claim that may be made by its manufacturer, is not guaranteed or endorsed by the publisher.
