# Supplementary material for: Prevalence, Clinical Features, and In-hospital Outcome of Fatty Liver Disease in Acute Aortic Dissection: A Single-Center Retrospective Study
Source: Front Cardiovasc Med. 2021 Aug 13;8:698285. doi: 10.3389/fcvm.2021.698285 (PMC8414544; doi:10.3389/fcvm.2021.698285)
Supplement: Supplementary file 1 [file Data_Sheet_1.docx]

Supplementary Material

# Supplementary table S.1

Table S.1. Univariate logistic regression analysis for complications of other main risk factors in acute aortic dissection.

|  |  | **Β** | **S.E.** | **Wald** | ***P*‐value** | **OR** | **95% CI** |
| --- | --- | --- | --- | --- | --- | --- | --- |
| Type A AAD |  |  |  |  |  |  |  |
| Age, linear |  |  |  |  |  |  |  |
| ACS |  | 0.015 | 0.019 | 0.625 | 0.4292 | 1.015 | 0.978-1.053 |
| Shock |  | 0.020 | 0.020 | 1.019 | 0.3127 | 1.020 | 0.981-1.061 |
| Renal dysfunction |  | -0.002 | 0.017 | 0.008 | 0.9273 | 0.998 | 0.966-1.032 |
| Anemia |  | 0.032 | 0.015 | 4.483 | 0.0342^*^ | 1.032 | 1.002-1.063 |
| Gender, male |  |  |  |  |  |  |  |
| ACS |  | 0.257 | 0.579 | 0.197 | 0.6573 | 1.293 | 0.416-4.023 |
| Shock |  | 0.016 | 0.589 | 0.001 | 0.9781 | 1.016 | 0.321-3.221 |
| Renal dysfunction |  | -0.006 | 0.497 | <0.001 | 0.9897 | 0.994 | 0.375-2.630 |
| Anemia |  | -1.415 | 0.388 | 13.293 | 0.0003^*^ | 0.243 | 0.114-0.520 |
| Alcohol consumption |  |  |  |  |  |  |  |
| ACS |  | -0.995 | 0.640 | 2.420 | 0.1198 | 0.370 | 0.106-1.295 |
| Shock |  | 0.461 | 0.496 | 0.862 | 0.3532 | 1.585 | 0.599-4.194 |
| Renal dysfunction |  | -0.158 | 0.466 | 0.115 | 0.7343 | 0.854 | 0.342-2.128 |
| Anemia |  | -0.812 | 0.449 | 3.268 | 0.0707 | 0.444 | 0.184-1.071 |
| Hyperlipidemia |  |  |  |  |  |  |  |
| ACS |  | -0.133 | 0.432 | 0.095 | 0.7575 | 0.875 | 0.376-2.040 |
| Shock |  | -0.182 | 0.467 | 0.153 | 0.6961 | 0.833 | 0.334-2.080 |
| Renal dysfunction |  | 0.697 | 0.401 | 3.012 | 0.0827 | 2.007 | 0.914-4.408 |
| Anemia |  | -0.463 | 0.343 | 1.823 | 0.1769 | 0.629 | 0.321-1.233 |
| Type B AAD |  |  |  |  |  |  |  |
| Age, linear |  |  |  |  |  |  |  |
| ACS |  | -0.036 | 0.053 | 0.466 | 0.4946 | 0.965 | 0.870-1.069 |
| Renal dysfunction |  | 0.007 | 0.021 | 0.121 | 0.7282 | 1.007 | 0.967-1.049 |
| Anemia |  | 0.020 | 0.018 | 1.120 | 0.2899 | 1.020 | 0.983-1.057 |
| Gender, male |  |  |  |  |  |  |  |
| ACS |  | - | - | - | 0.9982 | - | NA^1^ |
| Renal dysfunction |  | 0.044 | 0.662 | 0.004 | 0.9470 | 1.045 | 0.286-3.822 |
| Anemia |  | -0.532 | 0.515 | 1.067 | 0.3017 | 0.588 | 0.214-1.612 |
| Alcohol consumption |  |  |  |  |  |  |  |
| ACS |  | - | - | - | 0.9975 | - | NA^2^ |
| Renal dysfunction |  | 0.592 | 0.489 | 1.467 | 0.2258 | 1.807 | 0.694-4.709 |
| Anemia |  | -0.634 | 0.525 | 1.462 | 0.2266 | 0.530 | 0.190-1.483 |
| Hyperlipidemia |  |  |  |  |  |  |  |
| ACS |  | -0.802 | 1.234 | 0.423 | 0.5156 | 0.448 | 0.040-5.032 |
| Renal dysfunction |  | 0.117 | 0.475 | 0.060 | 0.8059 | 1.124 | 0.443-2.854 |
| Anemia |  | 0.693 | 0.438 | 2.507 | 0.1134 | 2.000 | 0.848-4.717 |

Abbreviation: AAD, Acute aortic dissection; ACS, Acute coronary syndrome; NA, not available.

1 All enlisted subjects with ACS in type B AAD were male.

2 All enlisted subjects with ACS in type B AAD were absent from alcohol consumption.

* p-value < 0.05

# Supplementary table S.2

Table S.2. Gender subgroups of AAD.

|  | Type A AD | | |  | Type B AD | | |  |
| --- | --- | --- | --- | --- | --- | --- | --- | --- |
| Gender | Male |  | Female |  | Male |  | Female |  |
| Characteristic | (N=155, %) |  | (N=37, %) | *p*-value | (N=158, %) |  | (N=29, %) | *p*-value |
| Age, Mean (SD), y | 50.5±11.4 |  | 58.2±10.2 | 0.0002^*^ | 54.5±11.7 |  | 58.3±8.6 | 0.1035 |
| Onset season |  |  |  |  |  |  |  |  |
| Spring | 35 (22.6) |  | 6 (16.2) | 0.7898 | 34 (21.5) |  | 8 (27.6) | 0.5060 |
| Summer | 27 (17.4) |  | 6 (16.2) |  | 27 (17.1) |  | 2 (6.9) |  |
| Autumn | 40 (25.8) |  | 12 (32.4) |  | 42 (26.6) |  | 7 (24.1) |  |
| Winter | 53 (34.2) |  | 13 (35.1) |  | 55 (34.8) |  | 12 (41.4) |  |
| Current smoker | 79 (51.0) |  | 0 (0) | <0.0001^*^ | 81 (51.3) |  | 4 (13.8) | 0.0002^*^ |
| Alcohol consumption | 47 (30.3) |  | 1 (2.7) | 0.0002^*^ | 53 (33.5) |  | 0 (0) | <0.0001^*^ |
| Fatty liver disease | 45 (29.0) |  | 3 (8.1) | 0.0100^*^ | 34 (21.5) |  | 2 (6.9) | 0.0755 |
| Medical condition |  |  |  |  |  |  |  |  |
| Hypertension | 131 (84.5) |  | 34 (91.9) | 0.3030 | 141 (89.2) |  | 23 (79.3) | 0.1356 |
| Diabetes Mellitus | 3 (1.9) |  | 5 (13.5) | 0.0074^*^ | 4 (2.5) |  | 4 (13.8) | 0.0212^*^ |
| Hyperlipidemia | 78 (50.3) |  | 12 (32.4) | 0.0662 | 84 (53.2) |  | 14 (48.3) | 0.6883 |
| Coronary heart disease | 16 (10.3) |  | 6 (16.2) | 0.3865 | 11 (7.0) |  | 4 (13.8) | 0.2573 |
| ACS | 21 (13.5) |  | 4 (10.8) | 0.7905 | 3 (1.9) |  | 0 (0) | 1.0000 |
| Shock | 17 (11.0) |  | 4 (10.8) | 1.0000 | 0 (0) |  | 0 (0) | - |
| SBP (mmHg) | 141.5±30.8 |  | 136.4±32.1 | 0.3617 | 160.2±27.0 |  | 153.4±27.7 | 0.2170 |
| DBP (mmHg) | 76.8±19.2 |  | 73.6±15.3 | 0.3382 | 89.5±15.8 |  | 91.0±15.6 | 0.6540 |
| BPD (mmHg) | 62.0 (50.0-76.0) |  | 60.0 (45.0-72.0) | 0.5089 | 70.0 (57.0-81.0) |  | 60.0 (51.0-75.0) | 0.0403^*^ |
| Biomarker |  |  |  |  |  |  |  |  |
| Wbc (10^9^/L) | 12.16 (10.48-14.67) |  | 11.27 (8.59-13.79) | 0.0471^*^ | 11.74±4.02 |  | 10.87±3.97 | 0.2840 |
| Hb (g/L) | 133.3±17.9 |  | 110.2±15.8 | <0.0001^*^ | 138.5 (126.0-147.0) |  | 119.0 (111.0-131.0) | <0.0001^*^ |
| Plt (10^9^/L) | 164.0 (135.0-189.0) |  | 146.0 (112.0-175.0) | 0.0178^*^ | 161.5 (130.0-191.0) |  | 162.0 (130.0-208.0) | 0.9792 |
| Alt (U/L) | 24.0 (16.0-36.0) |  | 20.0 (14.0-30.0) | 0.1702 | 20.0 (14.0-30.0) |  | 17.0 (13.0-28.0) | 0.3418 |
| Alb (g/L) | 39.38±3.86 |  | 38.40±2.98 | 0.1519 | 40.36±3.51 |  | 40.26±4.14 | 0.8893 |
| Tbil (μmol/L) | 16.61 (13.32-22.67) |  | 12.97 (9.34-17.69) | 0.0016^*^ | 16.18 (11.03-21.51) |  | 15.45 (9.46-20.20) | 0.2860 |
| Tch (mmol/L) | 3.93 (3.44-4.60) |  | 3.93 (3.61-4.87) | 0.7432 | 4.14 (3.64-4.78) |  | 4.52 (3.71-5.36) | 0.1374 |
| Tg (mmol/L) | 1.24 (0.89-1.89) |  | 1.07 (0.93-1.61) | 0.3475 | 1.08 (0.75-1.55) |  | 1.22 (0.70-1.71) | 0.7151 |
| Hdl (mmol/L) | 0.96 (0.84-1.13) |  | 1.06 (0.90-1.33) | 0.0244^*^ | 1.07 (0.87-1.34) |  | 1.24 (1.05-1.46) | 0.0298^*^ |
| Ldl (mmol/L) | 2.20±0.65 |  | 2.00±0.70 | 0.1064 | 2.34±0.71 |  | 2.31±0.84 | 0.8825 |
| Hs-CRP (mg/L) | 8.98 (3.34-33.11) |  | 5.08 (1.06-59.51) | 0.2174 | 6.09 (2.20-22.48) |  | 16.35 (3.43-60.61) | 0.0670 |
| Cr (μmol/L) | 93.0 (77.0-126.0) |  | 78.0 (58.0-107.0) | 0.0164^*^ | 83.0 (68.0-103.0) |  | 56.0 (44.0-65.0) | <0.0001^*^ |
| PT-INR | 1.05 (0.99-1.12) |  | 1.07 (0.98-1.16) | 0.4364 | 1.02 (0.96-1.08) |  | 1.01 (0.96-1.11) | 0.9006 |
| D-dimer (mg/L) | 5.54 (3.32-11.93) |  | 8.20 (4.01-15.14) | 0.1497 | 3.04 (1.79-7.12) |  | 4.18 (1.90-10.44) | 0.2274 |

Abbreviation: ACS, Acute coronary syndrome; SBP, Systolic blood pressure; DBP, Diastolic blood pressure; BPD, Blood pressure difference; Wbc, White blood cell counts; Hb, Hemoglobin; Plt, Platelets; Alt, Alanine aminotransferase; Ast, Aspartate aminotransferase; Alb, albumin; Tbil, Total bilirubin; Tch, Total cholesterol; Tg, Triglyceride; Hdl, High-density lipoprotein; Ldl, Low-density lipoprotein; Hs-CRP, High-sensitive C-reactive protein; Cr, Creatinine; PT-INR, prothrombin time-international normalized ratio

* p-value < 0.05

# Supplementary table S.3

Table S.3. Age subgroups of type A AD.

|  |  | Type A AD (n=192) | | | | | | | | |
| --- | --- | --- | --- | --- | --- | --- | --- | --- | --- | --- |
| Age group |  | ＜ 40 |  | 40-49 |  | 50-59 |  | ≥ 60 |  | *p*-value |
| Characteristic |  | (n=29, %) |  | (n=52, %) |  | (n=65, %) |  | (n=46, %) |  |  |
| Gender | Male | 28 (96.6) |  | 44 (84.6) |  | 53 (81.5) |  | 30 (65.2) |  | 0.0064^*^ |
|  | Female | 1 (3.4) |  | 8 (15.4) |  | 12 (18.5) |  | 16 (34.8) |  |  |
| Onset season | Spring | 4 (13.8) |  | 13 (25.0) |  | 14 (21.5) |  | 9 (19.6) |  | 0.8640 |
|  | Summer | 8 (27.6) |  | 7 (13.5) |  | 9 (13.8) |  | 9 (19.6) |  |  |
|  | Autumn | 6 (20.7) |  | 14 (26.9) |  | 20 (30.8) |  | 13 (28.3) |  |  |
|  | Winter | 11 (37.9) |  | 18 (34.6) |  | 22 (33.8) |  | 15 (32.6) |  |  |
| Current smoker |  | 14 (48.7) |  | 24 (46.2) |  | 29 (44.6) |  | 12 (26.1) |  | 0.1229 |
| Alcohol consumption |  | 9 (31.0) |  | 14 (26.9) |  | 21 (32.3) |  | 4 (8.7) |  | 0.0288^*^ |
| Fatty liver disease |  | 13 (44.8) |  | 15 (28.8) |  | 14 (21.5) |  | 6 (13.0) |  | 0.0154^*^ |
| Medical condition | Hypertension | 24 (82.8) |  | 42 (80.8) |  | 59 (90.8) |  | 40 (87.0) |  | 0.4248 |
|  | Diabetes Mellitus | 1 (3.4) |  | 2 (3.8) |  | 3 (4.6) |  | 2 (4.3) |  | 1.0000 |
|  | Hyperlipidemia | 17 (58.6) |  | 26 (50.0) |  | 28 (43.1) |  | 19 (41.3) |  | 0.4301 |
|  | CHD | 0 (0) |  | 2 (3.8) |  | 8 (12.3) |  | 12 (26.1) |  | 0.0009^*^ |
|  | ACS | 3 (10.3) |  | 7 (13.5) |  | 8 (12.3) |  | 7 (15.2) |  | 0.9619 |
|  | Shock | 2 (6.9) |  | 4 (7.7) |  | 11 (16.9) |  | 4 (8.7) |  | 0.3662 |
|  | SBP (mmHg) | 137.6±29.3 |  | 141.6±30.3 |  | 141.8±31.8 |  | 139.4±32.5 |  | 0.9271 |
|  | DBP (mmHg) | 76.3±20.8 |  | 77.0±17.7 |  | 75.3±17.3 |  | 76.0±19.9 |  | 0.9656 |
|  | BPD (mmHg) | 60.0 (50.0-70.0) |  | 60.0 (47.5-75.0) |  | 65.0 (50.0-81.0) |  | 59.5 (49.0-76.0) |  | 0.6542 |
| Biomarkers | Wbc (10^9^/L) | 13.73 (10.72-16.10) |  | 12.58 (11.16-15.12) |  | 12.04 (9.80-13.58) |  | 11.12 (8.59-13.63) |  | 0.0185^*^ |
|  | Hb (g/L) | 136.8±20.0 |  | 130.5±19.1 |  | 130.2±18.1 |  | 120.3±20.6 |  | 0.0030^*^ |
|  | Plt (10^9^/L) | 187.0 (160.5-205.0) |  | 166.0 (140.0-180.0) |  | 160.0 (128.5-177.5) |  | 139.5 (112.0-187.0) |  | 0.0047^*^ |
|  | Alt (U/L) | 31.0 (18.0-49.0) |  | 26.0 (19.0-35.0) |  | 22.0 (16.0-33.0) |  | 18.0 (13.0-35.0) |  | 0.0162^*^ |
|  | Ast (U/L) | 26.0 (22.5-38.5) |  | 29.0 (22.0-35.0) |  | 26.0 (20.5-39.5) |  | 27.5 (21.0-40.0) |  | 0.7120 |
|  | Alb (g/L) | 40.42±3.72 |  | 39.25±3.89 |  | 39.64±3.31 |  | 37.67±3.65 |  | 0.0079^*^ |
|  | Tbil (μmol/L) | 17.48 (10.38-22.76) |  | 15.42 (11.24-20.74) |  | 16.54 (12.84-20.58) |  | 15.83 (11.10-22.79) |  | 0.8277 |
|  | Tch (mmol/L) | 3.95±0.92 |  | 4.09±0.77 |  | 4.10±0.86 |  | 3.89±0.96 |  | 0.5648 |
|  | Tg (mmol/L) | 1.42 (0.95-2.36) |  | 1.49 (0.90-1.84) |  | 1.33 (0.94-1.87) |  | 1.00 (0.75-1.28) |  | 0.0183^*^ |
|  | Hdl (mmol/L) | 0.93 (0.84-1.02) |  | 0.99 (0.85-1.22) |  | 0.96 (0.86-1.12) |  | 1.04 (0.85-1.26) |  | 0.4790 |
|  | Ldl (mmol/L) | 2.13±0.63 |  | 2.25±0.74 |  | 2.20±0.59 |  | 2.02±0.67 |  | 0.3891 |
|  | Hs-CRP (mg/L) | 8.19 (5.50-11.30) |  | 10.90 (1.60-33.75) |  | 7.58 (1.20-40.28) |  | 8.34 (2.73-36.56) |  | 0.9017 |
|  | Cr (μmol/L) | 97.0 (83.5-132.5) |  | 89.0 (68.5-127.0) |  | 85.5 (75.0-117.0) |  | 87.0 (67.0-126.0) |  | 0.6551 |
|  | PT-INR | 1.04 (0.96-1.12) |  | 1.03 (0.99-1.11) |  | 1.05 (1.00-1.12) |  | 1.05 (0.98-1.19) |  | 0.8491 |
|  | D-dimer (mg/L) | 4.42 (2.10-9.58) |  | 5.95 (3.59-11.23) |  | 5.76 (3.38-10.92) |  | 8.50 (4.52-19.59) |  | 0.0445^*^ |

Abbreviation: CHD, Coronary heart disease; ACS, Acute coronary syndrome; SBP, Systolic blood pressure; DBP, Diastolic blood pressure; BPD, Blood pressure difference; Wbc, White blood cell counts; Hb, Hemoglobin; Plt, Platelets; Alt, Alanine aminotransferase; Ast, Aspartate aminotransferase; Alb, albumin; Tbil, Total bilirubin; Tch, Total cholesterol; Tg, Triglyceride; Hdl, High-density lipoprotein; Ldl, Low-density lipoprotein; Hs-CRP, High-sensitive C-reactive protein; Cr, Creatinine; PT-INR, prothrombin time-international normalized ratio

* p-value < 0.05

# Supplementary table S.4

Table S.4. Age subgroups of type B AD.

|  |  | Type B AD (n=192) | | | | | | | | |
| --- | --- | --- | --- | --- | --- | --- | --- | --- | --- | --- |
| Age group |  | ＜ 45 |  | 45-54 |  | 55-64 |  | ≥ 65 |  | *p*-value |
| Characteristic |  | (n=32, %) |  | (n=60, %) |  | (n=53, %) |  | (n=42, %) |  |  |
| Gender | Male | 30 (93.8) |  | 53 (88.3) |  | 40 (75.5) |  | 35 (83.3) |  | 0.1207 |
|  | Female | 2 (6.2) |  | 7 (11.7) |  | 13 (24.5) |  | 7 (16.7) |  |  |
| Onset season | Spring | 8 (25.0) |  | 10 (16.7) |  | 13 (24.5) |  | 11 (23.9) |  | 0.5817 |
|  | Summer | 5 (15.6) |  | 11 (18.3) |  | 10 (18.9) |  | 3 (7.1) |  |  |
|  | Autumn | 9 (28.1) |  | 17 (28.3) |  | 9 (17.0) |  | 14 (33.3) |  |  |
|  | Winter | 10 (31.2) |  | 22 (36.7) |  | 21 (39.6) |  | 14 (33.3) |  |  |
| Smoker |  | 11 (34.4) |  | 30 (50.0) |  | 27 (50.9) |  | 17 (40.5) |  | 0.3693 |
| Drinker |  | 6 (18.8) |  | 17 (28.3) |  | 14 (26.4) |  | 16 (38.1) |  | 0.3190 |
| Fatty liver disease |  | 11 (34.4) |  | 13 (21.7) |  | 10 (18.7) |  | 2 (4.8) |  | 0.0140^*^ |
| Medical condition | Hypertension | 31 (96.9) |  | 53 (88.3) |  | 45 (84.9) |  | 35 (83.3) |  | 0.2809 |
|  | Diabetes Mellitus | 0 (0) |  | 2 (3.3) |  | 4 (7.5) |  | 2 (4.8) |  | 0.4516 |
|  | Hyperlipidemia | 18 (56.2) |  | 34 (56.7) |  | 31 (58.5) |  | 15 (35.7) |  | 0.1066 |
|  | Coronary heart disease | 0 (0) |  | 5 (8.3) |  | 2 (3.8) |  | 8 (19.0) |  | 0.0139^*^ |
|  | Sleep apnea syndrome | 2 (6.2) |  | 2 (3.3) |  | 1 (1.9) |  | 0 (0) |  | 0.4391 |
|  | ACS | 1 (3.1) |  | 1 (1.7) |  | 1 (1.9) |  | 0 (0) |  | 0.8755 |
|  | SBP (mmHg) | 162.0 (140.0-175.0) |  | 160.0 (145.0-182.5) |  | 150.0 (140.0-170.0) |  | 164.0 (137.5-177.0) |  | 0.3037 |
|  | DBP (mmHg) | 93.5 (84.0-101.0) |  | 93.0 (80.0-102.5) |  | 85.0 (75.5-94.0) |  | 87.0 (80.0-95.5) |  | 0.0719 |
|  | BPD (mmHg) | 66.0 (51.0-77.0) |  | 69.5 (57.5-81.0) |  | 70.0 (58.0-80.0) |  | 69.0 (60.0-82.5) |  | 0.8007 |
| Biomarkers | Wbc (10^9^/L) | 12.93±3.66 |  | 12.17±3.81 |  | 11.17±4.31 |  | 10.34±3.83 |  | 0.0223^*^ |
|  | Hb (g/L) | 140.0 (136.0-147.0) |  | 141.0 (132.0-154.0) |  | 131.0 (115.0-144.5) |  | 130.0 (121.5-139.0) |  | 0.0203^*^ |
|  | Plt (10^9^/L) | 181.5 (141.0-206.0) |  | 157.0 (128.0-188.5) |  | 167.0 (145.0-208.0) |  | 157.0 (119.0-195.5) |  | 0.2100 |
|  | Alt (U/L) | 23.0 (18.0-53.0) |  | 22.0 (17.0-30.5) |  | 16.0 (13.0-28.5) |  | 17.0 (11.0-21.5) |  | 0.0006^*^ |
|  | Ast (U/L) | 25.0 (21.0-35.0) |  | 23.0 (21.0-29.5) |  | 22.0 (18.5-28.5) |  | 23.0 (18.0-29.5) |  | 0.5508 |
|  | Alb (g/L) | 41.60±3.68 |  | 40.71±3.47 |  | 40.39±3.39 |  | 38.82±3.59 |  | 0.0064^*^ |
|  | Tbil (μmol/L) | 16.74 (10.20-21.42) |  | 16.64 (11.46-24.25) |  | 15.45 (10.04-19.82) |  | 16.31 (10.76-20.70) |  | 0.7017 |
|  | Tch (mmol/L) | 4.30 (3.99-4.64) |  | 4.09 (3.64-4.92) |  | 4.59 (3.72-4.92) |  | 3.93 (3.44-4.48) |  | 0.0482^*^ |
|  | Tg (mmol/L) | 1.30 (0.86-2.61) |  | 1.20 (0.77-1.73) |  | 1.22 (0.66-1.64) |  | 0.89 (0.70-1.23) |  | 0.1746 |
|  | Hdl (mmol/L) | 0.92 (0.78-1.17) |  | 1.05 (0.90-1.32) |  | 1.09 (0.89-1.34) |  | 1.22 (0.98-1.44) |  | 0.0183^*^ |
|  | Ldl (mmol/L) | 2.28±0.62 |  | 2.34±0.77 |  | 2.55±0.75 |  | 2.10±0.67 |  | 0.0300^*^ |
|  | Hs-CRP (mg/L) | 5.49 (2.16-19.09) |  | 9.63 (2.25-33.82) |  | 5.12 (1.38-20.97) |  | 7.57 (3.00-61.61)) |  | 0.5240 |
|  | Cr (μmol/L) | 85.0 (68.0-102.0) |  | 76.5 (64.5-93.5) |  | 71.0 (59.5-84.5) |  | 84.0 (68.5-107.0) |  | 0.2305 |
|  | PT-INR | 1.04 (0.96-1.08) |  | 1.00 (0.95-1.04) |  | 1.02 (0.95-1.08) |  | 1.03 (0.96-1.08) |  | 0.3795 |
|  | D-dimer (mg/L) | 3.27 (1.91-4.34) |  | 3.72 (2.17-7.88) |  | 3.27 (1.56-6.58) |  | 2.66 (1.34-7.44) |  | 0.9829 |

Abbreviation: CHD, Coronary heart disease; ACS, Acute coronary syndrome; SBP, Systolic blood pressure; DBP, Diastolic blood pressure; BPD, Blood pressure difference; Wbc, White blood cell counts; Hb, Hemoglobin; Plt, Platelets; Alt, Alanine aminotransferase; Ast, Aspartate aminotransferase; Alb, albumin; Tbil, Total bilirubin; Tch, Total cholesterol; Tg, Triglyceride; Hdl, High-density lipoprotein; Ldl, Low-density lipoprotein; Hs-CRP, High-sensitive C-reactive protein; Cr, Creatinine; PT-INR, prothrombin time-international normalized ratio

* p-value < 0.05

# Supplementary table S.5

Table S.5. Drinker subgroups of AAD.

|  | Type A AD | | |  | Type B AD | | |  |
| --- | --- | --- | --- | --- | --- | --- | --- | --- |
| Alcohol consumption | Drinker |  | Non-drinker |  | Drinker |  | Non-drinker |  |
| Characteristic | (N=48, %) |  | (N=144, %) | *p*-value | (N=53, %) |  | (N=134, %) | *p*-value |
| Gender |  |  |  |  |  |  |  |  |
| Male | 47 (97.9) |  | 108 (75.0) | 0.0002^*^ | 53 (100.0) |  | 105 (78.4) | <0.0001^*^ |
| Female | 1 (2.1) |  | 36 (25.0) |  | 0 (0) |  | 29 (21.6) |  |
| Age, Mean (SD), y | 49.1±9.7 |  | 52.9±12.0 | 0.0267^*^ | 56.6±11.0 |  | 54.5±11.5 | 0.2740 |
| Onset season |  |  |  |  |  |  |  |  |
| Spring | 12 (25.0) |  | 29 (20.1) | 0.6427 | 13 (24.5) |  | 29 (21.6) | 0.9027 |
| Summer | 9 (18.8) |  | 24 (16.7) |  | 9 (17.0) |  | 20 (14.9) |  |
| Autumn | 14 (29.2) |  | 38 (26.4) |  | 14 (26.4) |  | 35 (26.1) |  |
| Winter | 13 (27.1) |  | 53 (36.8) |  | 17 (32.1) |  | 50 (37.3) |  |
| Current smoker | 38 (79.2) |  | 41 (28.5) | <0.0001^*^ | 41 (77.4) |  | 44 (32.8) | <0.0001^*^ |
| Fatty liver disease | 18 (37.5) |  | 30 (20.8) | 0.0330^*^ | 12 (22.6) |  | 24 (17.9) | 0.5373 |
| Medical condition |  |  |  |  |  |  |  |  |
| Hypertension | 42 (87.5) |  | 123 (85.4) | 0.8144 | 50 (94.3) |  | 114 (85.1) | 0.0899 |
| Diabetes Mellitus | 2 (4.2) |  | 6 (4.2) | 1.0000 | 1 (1.9) |  | 7 (5.2) | 0.4441 |
| Hyperlipidemia | 28 (58.3) |  | 62 (43.1) | 0.0943 | 27 (50.9) |  | 71 (53.0) | 0.8714 |
| Coronary heart disease | 3 (6.3) |  | 19 (13.2) | 0.2945 | 5 (9.4) |  | 10 (7.5) | 0.7657 |
| ACS | 3 (6.3) |  | 22 (15.3) | 0.1389 | 0 (0) |  | 3 (2.2) | 0.5596 |
| Shock | 7 (14.6) |  | 14 (9.7) | 0.4226 | 0 (0) |  | 0 (0) | - |
| SBP (mmHg) | 144.6±31.6 |  | 139.2±30.8 | 0.2959 | 166.1±27.8 |  | 156.4±26.5 | 0.0266^*^ |
| DBP (mmHg) | 79.3±20.6 |  | 75.2±17.8 | 0.1856 | 94.4±15.6 |  | 87.9±15.4 | 0.0101^*^ |
| BPD (mmHg) | 68.5 (49.5-75.0) |  | 60.0 (49.0-76.5) | 0.4714 | 70.0 (60.0-82.0) |  | 68.5 (54.0-80.0) | 0.2703 |
| Biomarker |  |  |  |  |  |  |  |  |
| Wbc (10^9^/L) | 12.59 (11.31-14.12) |  | 11.76 (9.50-14.62) | 0.2180 | 11.96±3.57 |  | 11.47±4.18 | 0.4532 |
| Hb (g/L) | 136.7±16.8 |  | 126.3±20.0 | 0.0081^*^ | 139.0 (131.0-148.0) |  | 135.5 (121.0-144.0) | 0.0268^*^ |
| Plt (10^9^/L) | 174.5 (153.0-208.0) |  | 155.0 (127.0-180.5) | 0.0032^*^ | 160.0 (127.0-186.0) |  | 164.0 (132.0-205.0) | 0.3692 |
| Alt (U/L) | 25.0 (16.5-38.0) |  | 23.0 (15.0-35.0) | 0.2558 | 19.0 (13.0-24.0) |  | 20.5 (14.0-32.0) | 0.1511 |
| Alb (g/L) | 40.07±3.55 |  | 38.90±3.74 | 0.0624 | 40.45±3.36 |  | 40.31±3.71 | 0.8096 |
| Tbil (μmol/L) | 16.44 (14.24-22.38) |  | 15.85 (11.28-21.41) | 0.2135 | 18.48 (13.78-24.02) |  | 15.39 (10.18-19.75) | 0.0060^*^ |
| Tch (mmol/L) | 4.11 (3.86-4.84) |  | 3.85 (3.30-4.58) | 0.0082^*^ | 4.18 (3.80-4.98) |  | 4.14 (3.58-4.73) | 0.1721 |
| Tg (mmol/L) | 1.44 (1.06-1.87) |  | 1.10 (0.83-1.80) | 0.1046 | 1.19 (0.81-1.58) |  | 1.06 (0.72-1.55) | 0.3920 |
| Hdl (mmol/L) | 1.04 (0.90-1.21) |  | 0.96 (0.84-1.15) | 0.1231 | 1.28 (1.00-1.50) |  | 1.05 (0.87-1.26) | 0.0052^*^ |
| Ldl (mmol/L) | 2.36±0.63 |  | 2.09±0.66 | 0.0155^*^ | 2.39±0.85 |  | 2.31±0.68 | 0.5494 |
| Hs-CRP (mg/L) | 8.84 (3.34-20.63) |  | 8.09 (1.70-37.56) | 0.9095 | 9.27 (2.51-41.03) |  | 6.08 (2.28-27.37) | 0.4483 |
| Cr (μmol/L) | 100.0 (80.5-119.0) |  | 87.0 (71.5-126.0) | 0.1623 | 85.0 (71.0-107.0) |  | 75.5 (60.0-99.0) | 0.0311^*^ |
| PT-INR | 1.04 (0.95-1.11) |  | 1.05 (0.99-1.14) | 0.1997 | 1.04 (0.96-1.08) |  | 1.01 (0.96-1.08) | 0.5567 |
| D-dimer (mg/L) | 5.22 (3.44-9.14) |  | 6.38 (3.34-14.26) | 0.1923 | 3.84 (1.78-7.68) |  | 3.08 (1.87-7.32) | 0.5395 |

Abbreviation: ACS, Acute coronary syndrome; SBP, Systolic blood pressure; DBP, Diastolic blood pressure; BPD, Blood pressure difference; Wbc, White blood cell counts; Hb, Hemoglobin; Plt, Platelets; Alt, Alanine aminotransferase; Ast, Aspartate aminotransferase; Alb, albumin; Tbil, Total bilirubin; Tch, Total cholesterol; Tg, Triglyceride; Hdl, High-density lipoprotein; Ldl, Low-density lipoprotein; Hs-CRP, High-sensitive C-reactive protein; Cr, Creatinine; PT-INR, prothrombin time-international normalized ratio

* p-value < 0.05

# Supplementary table S.6

Table S.6. Hyperlipidemia subgroups of AAD.

|  | Type A AD | | |  | Type B AD | | |  |
| --- | --- | --- | --- | --- | --- | --- | --- | --- |
| Hyperlipidemia | Presence |  | Absence |  | Presence |  | Absence |  |
| Characteristic | (N=90, %) |  | (N=102, %) | *p*-value | (N=98, %) |  | (N=89, %) | *p*-value |
| Gender |  |  |  |  |  |  |  |  |
| Male | 78 (86.7) |  | 77 (75.5) | 0.0662 | 84 (85.7) |  | 74 (83.1) | 0.6883 |
| Female | 12 (13.3) |  | 25 (24.5) |  | 14 (14.3) |  | 15 (16.9) |  |
| Age, Mean (SD), y | 50.7±11.8 |  | 53.1±11.3 | 0.1532 | 53.6±10.1 |  | 56.8±12.4 | 0.0614 |
| Onset season |  |  |  |  |  |  |  |  |
| Spring | 21 (23.3) |  | 20 (19.6) | 0.2319 | 22 (22.4) |  | 20 (22.5) | 0.7648 |
| Summer | 17 (18.9) |  | 16 (15.7) |  | 17 (17.3) |  | 12 (13.5) |  |
| Autumn | 18 (20.0) |  | 34 (33.3) |  | 27 (27.6) |  | 22 (24.7) |  |
| Winter | 34 (37.8) |  | 32 (31.4) |  | 32 (32.7) |  | 35 (39.3) |  |
| Current smoker | 43 (47.8) |  | 36 (35.3) | 0.1058 | 44 (44.9) |  | 41 (46.1) | 0.8842 |
| Alcohol consumption | 28 (31.1) |  | 20 (19.6) | 0.0943 | 27 (27.6) |  | 26 (29.2) | 0.8714 |
| Fatty liver disease | 32 (35.6) |  | 16 (15.7) | 0.0024^*^ | 26 (26.5) |  | 10 (11.2) | 0.0093^*^ |
| Medical condition |  |  |  |  |  |  |  |  |
| Hypertension | 78 (86.7) |  | 87 (85.3) | 0.8374 | 86 (87.8) |  | 78 (87.6) | 1.0000 |
| Diabetes Mellitus | 7 (7.8) |  | 1 (1.0) | 0.0268^*^ | 4 (4.1) |  | 4 (4.5) | 1.0000 |
| Coronary heart disease | 7 (7.8) |  | 15 (14.7) | 0.1738 | 7 (7.1) |  | 8 (9.0) | 0.7889 |
| ACS | 11 (12.2) |  | 14 (13.7) | 0.8317 | 1 (1.0) |  | 2 (2.2) | 0.6056 |
| Shock | 9 (10.0) |  | 12 (11.8) | 0.8180 | 0 (0) |  | 0 (0) | - |
| SBP (mmHg) | 145.4±31.2 |  | 136.2±30.4 | 0.0388^*^ | 159.7±23.2 |  | 158.4±31.1 | 0.7506 |
| DBP (mmHg) | 78.1±18.0 |  | 74.5±18.9 | 0.1825 | 91.2±15.0 |  | 88.2±16.4 | 0.2004 |
| BPD (mmHg) | 66.0 (50.0-80.0) |  | 60.0 (46.0-72.0) | 0.0513 | 68.5 (57.0-80.0) |  | 70.0 (55.0-81.0) | 0.7556 |
| Biomarker |  |  |  |  |  |  |  |  |
| Wbc (10^9^/L) | 12.67 (10.93-15.86) |  | 11.70 (9.40-13.71) | 0.0139^*^ | 11.66±3.83 |  | 11.55±4.22 | 0.8598 |
| Hb (g/L) | 131.0±19.9 |  | 127.0±19.6 | 0.1660 | 139.0 (123.0-147.0) |  | 136.0 (123.0-143.0) | 0.2994 |
| Plt (10^9^/L) | 166.0 (141.0-189.0) |  | 157.0 (128.0-180.0) | 0.0748 | 169.0 (135.0-206.0) |  | 157.0 (125.0-191.0) | 0.1179 |
| Alt (U/L) | 25.0 (16.5-45.0) |  | 22.0 (15.0-32.0) | 0.0258^*^ | 21.0 (15.0-30.0) |  | 18.0 (13.0-27.0) | 0.0884 |
| Alb (g/L) | 39.72±4.27 |  | 38.74±3.12 | 0.0746 | 40.51±3.53 |  | 40.17±3.69 | 0.5190 |
| Tbil (μmol/L) | 15.98 (11.01-20.83) |  | 16.28 (12.72-22.38) | 0.3254 | 16.40 (10.92-21.42) |  | 15.96 (10.60-20.80) | 0.9452 |
| Tch (mmol/L) | 4.17 (3.70-5.08) |  | 3.82 (3.25-4.20) | <0.0001^*^ | 4.54 (3.79-5.42) |  | 4.00 (3.56-4.40) | <0.0001^*^ |
| Tg (mmol/L) | 1.89 (1.14-2.50) |  | 1.00 (0.74-1.34) | <0.0001^*^ | 1.48 (0.91-2.53) |  | 0.84 (0.64-1.21) | <0.0001^*^ |
| Hdl (mmol/L) | 0.90 (0.76-1.10) |  | 1.04 (0.93-1.20) | <0.0001^*^ | 1.04 (0.81-1.25) |  | 1.20 (1.00-1.42) | 0.0002^*^ |
| Ldl (mmol/L) | 2.32±0.75 |  | 2.02±0.58 | 0.0026^*^ | 2.52 (1.95-3.12) |  | 2.09 (1.80-2.48) | <0.0001^*^ |
| Hs-CRP (mg/L) | 8.24 (3.37-33.11) |  | 8.38 (1.38-40.04) | 0.6900 | 7.83 (2.51-25.59) |  | 6.08 (2.09-39.84) | 0.7957 |
| Cr (μmol/L) | 99.5 (79.0-129.5) |  | 85.0 (69.0-118.0) | 0.0222^*^ | 77.0 (63.0-99.0) |  | 82.0 (65.0-102.0) | 0.4659 |
| PT-INR | 1.04 (0.98-1.15) |  | 1.05 (0.98-1.13) | 0.9815 | 0.99 (0.94-1.06) |  | 1.04 (0.98-1.10) | 0.0004^*^ |
| D-dimer (mg/L) | 6.28 (3.50-14.23) |  | 5.34 (2.98-11.08) | 0.2367 | 2.82 (1.75-5.96) |  | 3.27 (2.12-7.90) | 0.1392 |

Abbreviation: ACS, Acute coronary syndrome; SBP, Systolic blood pressure; DBP, Diastolic blood pressure; BPD, Blood pressure difference; Wbc, White blood cell counts; Hb, Hemoglobin; Plt, Platelets; Alt, Alanine aminotransferase; Ast, Aspartate aminotransferase; Alb, albumin; Tbil, Total bilirubin; Tch, Total cholesterol; Tg, Triglyceride; Hdl, High-density lipoprotein; Ldl, Low-density lipoprotein; Hs-CRP, High-sensitive C-reactive protein; Cr, Creatinine; PT-INR, prothrombin time-international normalized ratio

* p-value < 0.05

# Supplementary Figures S.1


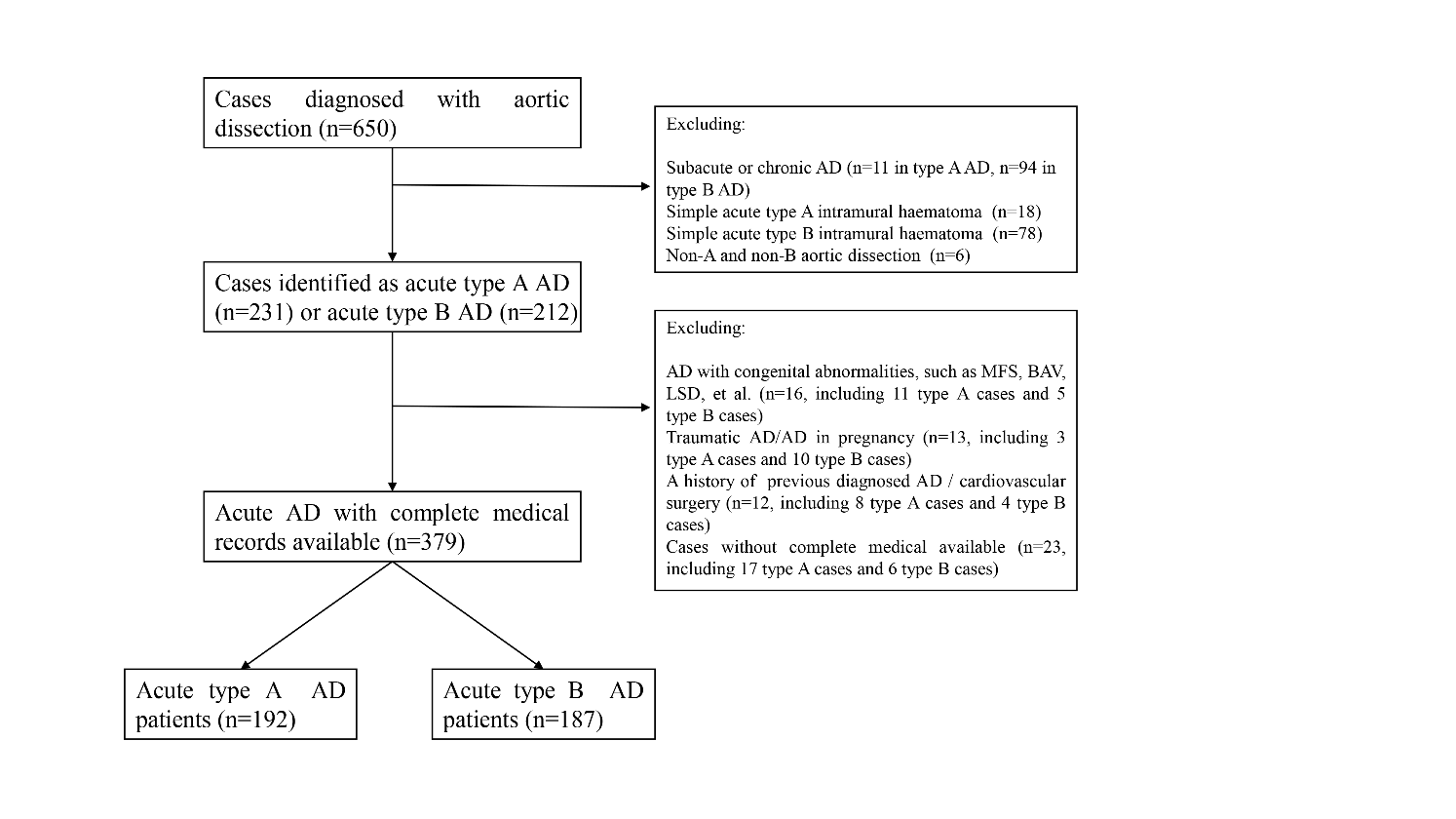


**Supplementary Figure S.1.** The flowchart of study population selection. MFS, Marfan syndrome, BAV, bicuspid aortic valve; LDS, Loeys-Dietz syndrome.

# Supplementary Figure S.2.

The propensity of each patient was obtained from a logistic regression model. To reduce potential confounding all baseline characteristics such as age, gender, etc., were included as covariates in the model. Covariates were selected based on their distribution among FLD and non-FLD groups. Final covariates for type A AD were age, gender, current smoker, alcohol consumption, diabetes mellitus, hypertension, hyperlipidemia, CHD, ACS, and hypertrophic cardiomyopathy. For type B AD, the final covariates were age, gender, current smoker, alcohol consumption, hypertension, hyperlipidemia, CHD, hypertrophic cardiomyopathy, and anemia. AAD cases with FLD were matched in a 1:2 ratio to cases without fatty liver based on the propensity score with a standard caliper width of 0.2.

Dot plot demonstrate the distribution of propensity scores for type A acute aortic dissection patients. Stratified into matched and unmatched categories.


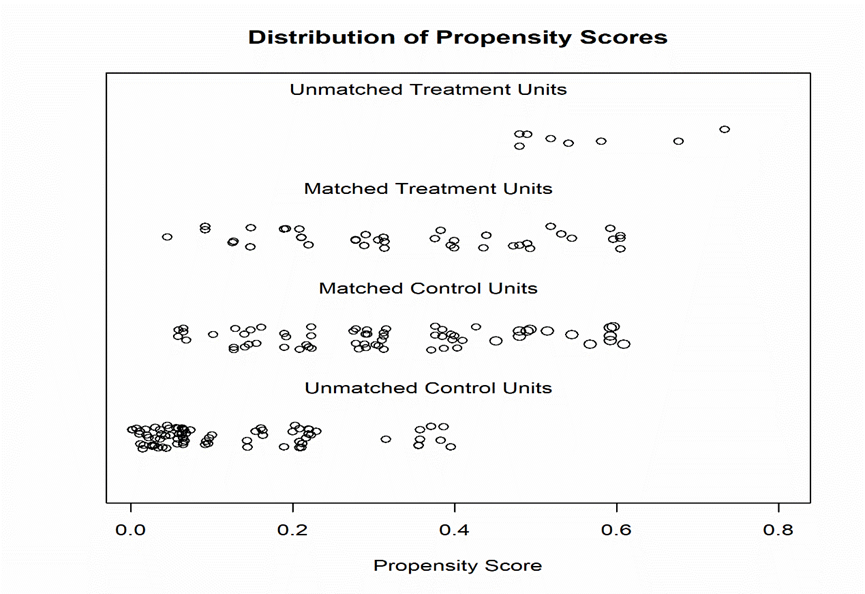


**Supplementary Figure S.2.** Propensity score distribution among type A acute aortic dissection.

# 5 Supplementary Figure S.3


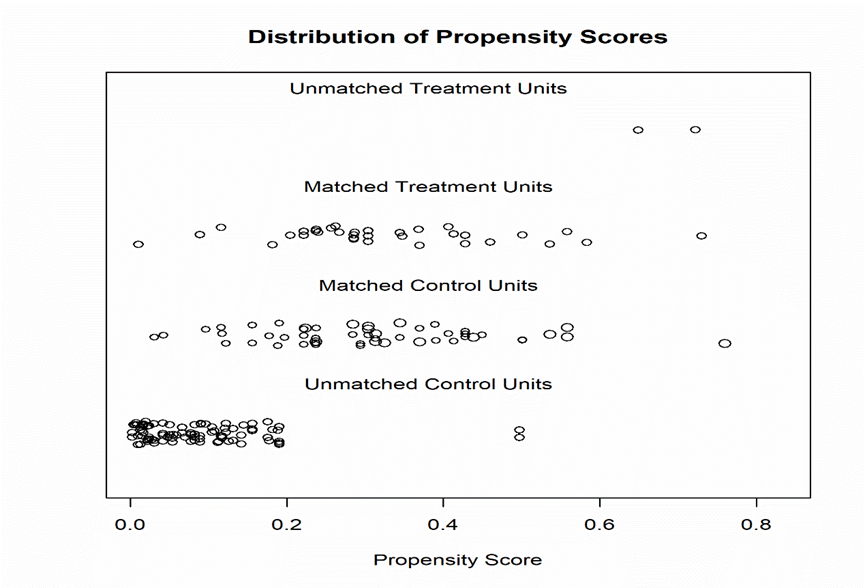


**Supplementary Figure S.3.** Propensity score distribution among type B acute aortic dissection.
